# Current Bioinformatics Tools in Precision Oncology

**DOI:** 10.1002/mco2.70243

**Published:** 2025-07-09

**Authors:** Tesfaye Wolde, Vipul Bhardwaj, Vijay Pandey

**Affiliations:** ^1^ Institute of Biopharmaceutical and Health Engineering Tsinghua Shenzhen International Graduate School Tsinghua University Shenzhen China; ^2^ Tsinghua Shenzhen International Graduate School Tsinghua University Shenzhen China

**Keywords:** bioinformatics, biomarkers, multiomic, oncotherapeutics, precision oncology

## Abstract

Integrating bioinformatics tools has profoundly transformed precision oncology by identifying essential molecular targets for personalized treatment. The rapid development of high‐throughput sequencing and multiomics technologies creates complex datasets that require robust computational methods to extract meaningful insights. Nonetheless, the clinical application of multiomics data continues to pose significant challenges. This review explores advanced bioinformatics tools utilized within multiomics, emphasizing their pivotal role in discovering cancer biomarkers. Cloud‐based platforms, such as Galaxy and DNAnexus, facilitate streamlined data processing, while single‐cell analysis software, including Seurat, identifies rare cellular subpopulations. Further integration of artificial intelligence with machine learning approaches improves predictive modeling and diagnostic accuracy. Spatial omics technologies correlate molecular signatures within tumor microenvironments, guiding treatment strategies. Bioinformatics integrates these technologies to establish a new standard in precision oncology, thereby enhancing therapy efficacy. Collaborative initiatives between The Cancer Genome Atlas and cBioPortal expedite advancements through the sharing open data and implementing standardized methodologies. Advancing multiomics integration techniques alongside improved computational capabilities is essential for discovering new biomarkers and refining precision medicine strategies. Future efforts should focus on merging multiomics techniques with innovative computational methods to drive novel biomarker discovery and improve precision medicine applications.

## Graphical abstract

This framework illustrates the comprehensive integration of bioinformatics tools to enhance biomarker and therapeutic target discovery. It incorporates multiomics data—spanning RNA, DNA, proteins, lipids, and chromatin—alongside preclinical (in vitro, ex vivo, and in vivo) and clinical (clinical trials) validation approaches. By leveraging advanced computational techniques, including artificial intelligence (AI), machine learning (ML), and deep learning (DL), this workflow enables precise detection, classification, and prediction of disease‐associated biomarkers. This integrative strategy supports diagnostic and prognostic screening, facilitates biomarker discovery, and aids in identifying potential therapeutic targets, ultimately advancing precision medicine and innovative treatment strategies.

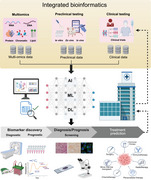

## 1 Introduction

Oncology is swiftly evolving from a generic, one‐size‐fits‐all treatment model to a personalized approach rooted in precision medicine [1, 2]. This evolution is driven by advancements in molecular biology, high‐throughput sequencing, and computational tools that help integrate complex multiomics data effectively [3]. Precision oncology aims to customize treatments for individual patients, similar to how fingerprints reflect genetic, epigenetic, and environmental identities, enabling personalized strategies [4]. This approach is central to identifying and validating biomarkers that signify measurable events associated with cancer onset, progression, and therapeutic response. Biomarkers can arise from various sources, including tumor tissues, blood, and other bodily fluids, encompassing DNA, RNA, proteins, and metabolites [5, 6, 7, 8]. Leveraging these biomarkers can significantly improve patient outcomes through early diagnosis, risk assessment, treatment selection, and disease monitoring. For instance, specific mutations in the EGFR gene are used as indicators for targeted therapies in non‐small cell lung cancer (NSCLC), guiding the use of EGFR inhibitors [9, 10]. Moreover, epigenetic biomarkers, which influence gene expression without altering the underlying DNA sequence, play a crucial role in cancer development [11]. These include epigenetic modifications such as noncoding RNA profiles, histone changes, and DNA methylation patterns that can act as cancer biomarkers. Notably, hypermethylation and silencing of tumor suppressor gene promoters, like MLH*1* in various cancers, may drive tumor growth and serve as potential early indicators for diagnosis or response to therapy, assessing PD‐L1 expression levels is also essential for determining candidacy for immunotherapy [12, 13]. Other currently used biomarkers include breast cancer gene 1/2 (*BRCA* 1/2) mutations, which signal suitability for PARP inhibitor treatment [14]. However, the discovery and validation of reliable biomarkers pose significant challenges due to the intricate nature of cancer biology and variability within and among tumors.

Recent advancements in bioinformatics have rapidly transformed precision oncology, leading to patient‐centered treatment strategies based on molecular biomarkers [15]. Bioinformatics, which intersects biology, computer science, and mathematics for biological data analysis, has gained medical relevance in cancer research with the introduction of high‐throughput techniques like next‐generation sequencing (NGS), microarrays, and proteomics [16, 17]. The enormous data generated by these technologies can be overwhelming without suitable analytical methods. Bioinformatics tools help uncover patterns, correlations, and anomalies that could signify potential biomarkers. For instance, Wolde et al. [18] applied an integrated bioinformatics strategy to identify a novel signature of nine *immune‐related genes* as potential biomarkers and therapeutic targets in ovarian carcinoma. In another study, Zhao et al. [19] discovered and validated a seven‐gene signature (*AFAP1L2*, *CAMK1D*, *LOXL2*, *PIK3CG*, *PLEKHG1*, *RARRES2*, and *SPP1*) for prognosis stratification in advanced lung adenocarcinoma patients, discussing its potential to predict survival outcomes. Additionally, Snijesh et al. [20] utilized data from The Cancer Genome Atlas (TCGA) to categorize endometrial cancer (EC) tumors by sonic hedgehog (SHH) pathway activation. They found that high SHH tumors display a less aggressive phenotype, lower mutational burden, and improved survival outcomes, underlining the prognostic importance of SHH signaling in EC [20]. The integration of bioinformatics methods, statistical modeling, and network analysis could deepen our understanding of cancer biology and lead to the development of predictive tools.

In precision oncology, biomarker discovery relies on an array of bioinformatics tools that manage and analyze complex data. For example, data pipelines and specialized software are used to identify *differentially expressed genes* (DEGs), predict patient outcomes, and simulate treatment responses [16]. Genomic analysis toolkits such as the Genome Analysis Toolkit (GATK), Spliced Transcript Alignment to a Reference (STAR), and HISAT2 work together to process sequencing data, while DESeq2 and EdgeR focus on detecting differential gene expression in RNA sequencing (RNA‐seq) [21]. Additionally, proteomic analysis tools like MaxQuant and Proteome Discoverer facilitate the quantification and identification of proteins to uncover potential molecular biomarkers [22]. Integrative platforms, including cBioPortal and Oncomine, combine multiomic datasets, providing a comprehensive perspective on tumor biology and aiding researchers in their search for promising biomarkers across various tumors [23, 24].

Advancements in biomarker identification methods and the growth of extensive data repositories have increased data complexity and volume, necessitating sophisticated analytical techniques [25]. This has increased the need for machine learning (ML) and predictive algorithms. ML algorithms are designed to handle and analyze large, high‐dimensional datasets, and molecular networks in network medicine, revealing patterns and relationships often overlooked by traditional methods [26]. With the expansion of artificial intelligence (AI), various ML frameworks, including Python's scikit‐learn, TensorFlow, and Keras, are now utilized for predictive modeling in oncology [27]. These tools can examine historical patient data to forecast outcomes and treatment responses based on recognized biomarkers. Additionally, network and pathway analysis tools like STRING and Cytoscape investigate molecular interactions and frequently regulated biological pathways that connect and influence tumor behavior through biomarkers [28]. Clinical data integration is also experiencing growth, with software developed to merge clinical data with molecular profiles. Platforms such as REDCap and OpenClinica facilitate collecting and analyzing clinical outcomes in conjunction with genomic and proteomic data [29].

Although advancements in bioinformatics tools have accelerated biomarker discovery, several challenges remain, including data acquisition, reproducibility, quality control, interoperability among various platforms, and inconsistent reporting concerns [30]. A multidisciplinary approach that includes clinical and ethical expertise is essential for effectively applying bioinformatics tools in diagnosis, preventive medicine, and personalized therapeutic strategies. The extensive and complex datasets generated by high‐throughput technologies, particularly NGS methods, necessitate the use of bioinformatics tools for their analysis [31]. The adoption of bioinformatics and related tools is transforming precision oncology by enabling swift, comprehensive genomic data analysis, evaluating disease mechanisms, and facilitating the identification of potential biomarkers for customized therapies. Moreover, the complexity and diversity of cancer present significant obstacles to identifying universally applicable biomarkers. Nonetheless, these challenges create opportunities for continued innovation in bioinformatics, which can radically enhance the analytical process. Progress in AI and ML predictive algorithms is revealing patterns and managing large datasets that exceed human analytical capabilities. Additionally, the increasing focus on multiomics approaches, integrating genomics, transcriptomics, proteomics, and metabolomics, provides a comprehensive perspective for understanding the fundamental mechanisms of cancer [32]. Collectively, these advancements hold the potential to address existing challenges and limitations in bioinformatics, transforming complex data into actionable strategies for precision‐driven care.

This review provides an in‐depth overview of how bioinformatics tools integrate and transform biomarker discovery and therapeutic strategies in precision oncology. We discuss their ability to identify molecular targets through the integration and analysis of multiomics data. Additionally, we highlight several significant bioinformatics platforms, including AI and ML‐driven predictive tools, as well as emerging technologies like spatial omics, and discuss their roles in cancer diagnosis, prognosis, and treatment optimization. Furthermore, we address challenges related to data interpretation and clinical translation, along with ethical implications, stressing the importance of interdisciplinary collaboration and open data‐sharing initiatives. This review aims to bridge bioinformatics and clinical oncology, creating new opportunities to utilize these computational advancements for enhancing patient‐specific therapeutic strategies and achieving success in precision cancer medicine.

## 2 Types of Omics Data in Biomarker Discovery

**FIGURE 1:**
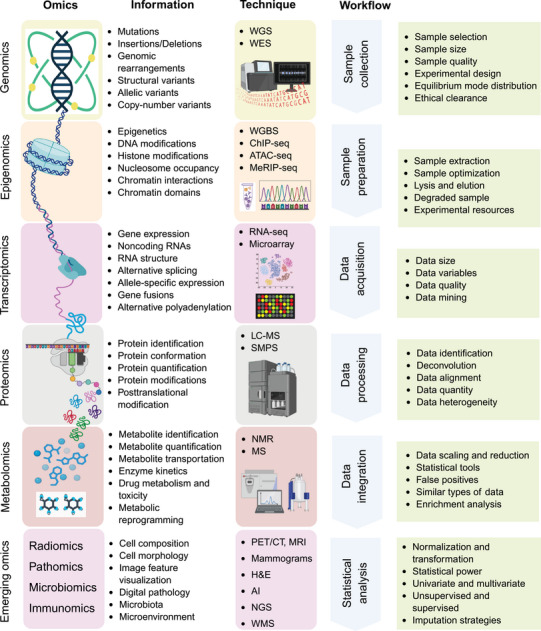
This comprehensive figure displays the complex landscape of omics technologies and their roles in biological research. Multiomics technologies have emerged to profile genome sequences, epigenetic features, gene expression, protein levels, metabolite abundances, and more. The illustration clarifies the types of insights offered by omics, such as genetic variations and epigenetic changes. It also details essential techniques like whole‐genome sequencing (WGS), whole‐exome sequencing (WES), and advanced methods including whole genome bisulfite sequencing (WGBS), chromatin immunoprecipitation sequencing (ChIP‐seq), and assay for transposase‐accessible chromatin using sequencing (ATAC‐seq) for examining chromatin dynamics. Additionally, it emphasizes the workflow from sample collection and preparation to data acquisition, pointing out challenges related to sample quality, experimental design, and ethical issues. The figure also addresses difficulties faced during the workflow. Last, it presents the emerging omics and the involvement of AI in deciphering complex datasets, highlighting the significance of multiomics integration in promoting cancer research and personalized medicine. The figure is generated using BioRender.com. NGS: next‐generation sequencing, ChIP‐seq: chromatin immunoprecipitation sequencing, ATAC‐seq: assay for transposase‐accessible chromatin sequencing, *RNA‐seq*: *RNA sequencing*, LC–MS: liquid chromatography–mass spectrometry, SIMP: stable isotope mass profiling, 2D‐DIGE: two‐dimensional difference gel electrophoresis, SRM: selected reaction monitoring, NMR: nuclear magnetic resonance, AI: artificial intelligence, ML: machine learning, WGCNA: weighted gene coexpression network analysis, GSEA: gene set enrichment analysis, TCGA: the cancer genome atlas, GEO: Gene Expression Omnibus, DDBJ: DNA Data Bank of Japan, GEPIA: gene expression profiling interactive analysis, GenBank: genetic sequence database, DNAnexus: cloud‐based platform for data analysis, COSMIC: catalogue of somatic mutations in cancer, *CA‐125*: cancer antigen 125, KRAS G12V: mutation of the *KRAS gene at codon 12* (glycine to valine), FGFR3: fibroblast growth factor receptor 3, MGMT: O‐6‐methylguanine‐DNA methyltransferase, TP53: tumor protein 53, microRNA: small, noncoding RNA molecules (e.g., *miR‐21, miR‐155*), *lncRNA*: long noncoding RNA, PVT1: plasmacytoma variant translocation 1.

The discovery and validation of biomarkers in precision oncology primarily relies on omics technologies, which facilitate the comprehensive analysis of biological molecules across diverse contexts [33]. The term “Omics” refers to various biological studies aimed at the extensive characterization of biomolecules, such as genomes, transcriptomes, proteomes, and metabolomes (Figure 1). The various types of omics, which include genomics, transcriptomics, proteomics, epigenomics, and metabolomics data, offer complementary insights that enhance our understanding of cancer biology, help identify potential biomarkers, and aid in patient stratification populations [34]. As technological advancements progress, omics technologies are set to play an increasingly vital role in precision oncology, raising innovation in biomarker discovery and personalized medicine. This section offers a comprehensive summary of the different omics data types and the bioinformatics tools utilized for their analysis.

### 2.1 Genomics

Genomics constitutes a comprehensive examination of an organism's genome, involving the sequencing, mapping, and analysis of its DNA [35]. Advancements in technology related to DNA sequencing, particularly NGS, facilitate extensive analysis of entire genomes or specific regions, thereby enabling the identification of mutations such as single‐nucleotide polymorphisms (SNPs), insertions, deletions, copy number variations (CNVs), and structural modifications that are crucial to the development of cancer [36]. Techniques including whole‐genome sequencing (WGS), single‐cell DNA sequencing, and targeted gene panels empower researchers to precisely identify mutations that can inform therapeutic strategies. For instance, mutations in genes such as KRAS, BRAF, and TP53 correlate with specific types of cancer and therapeutic responses, underscoring the clinical significance of genomic profiling [37, 38]. The intricate nature of genomic data requires sophisticated bioinformatics support for comprehensive analysis. Specialized computational platforms are indispensable for the interpretation of variants. Bioinformatics tools, such as GATK, are recognized as leading solutions for the detection of SNPS and small insertions and deletions, while MuTect specializes in identifying somatic mutations in paired tumor‐normal samples [39, 40]. ANNOVAR enhances these methodologies by offering detailed variant annotations based on functional consequences [41]. Moreover, single‐cell genomics tools, such as CellRanger, have revolutionized gene expression analysis at the individual cell level, essential for understanding cancer heterogeneity and evolution [42]. Tools like MORPHEUS and Monocle 3 facilitate interactive clustering of gene expression, lineage tracing, and trajectory analysis, all of which are vital for unraveling cancer progression and cellular differentiation [43, 44]. By integrating these bioinformatics instruments to convert raw genetic data from DNA sequencing techniques into meaningful clinical insights, researchers can uncover the prognostic and predictive value of specific genetic alterations, ultimately advancing personalized approaches in oncology.

### 2.2 Transcriptomics

Transcriptomics provides an ongoing perspective on gene expression in cells, offering vital insights into the molecular mechanisms underlying cancer development and progression [45]. Transcriptomics focuses on analyzing RNA transcripts, such as mRNA, noncoding RNA, and miRNA, to assess gene expression variances between cancerous and healthy tissues. Advanced technologies like RNA‐seq and microarrays have reinvented gene expression profiling, revealing patterns associated with different tumor types, stages, and responses to treatment [46, 47]. For example, the high and low expression of certain oncogenes and tumor suppressor genes respectively can serve as diagnostic or prognostic biomarkers, enhancing our understanding and potentially aiding in mitigating disease progression [48, 49].

Bioinformatics tools play a vital role in analyzing transcriptomic data [50]. Differential gene expression analysis is key for identifying genes that are either upregulated or downregulated in cancer, supported by statistical model‐based tools such as DESeq2 and EdgeR that work with count‐based RNA‐seq data [21, 51]. While limma was originally created for microarray research, it is now commonly used in RNA‐seq to uncover significant expression patterns [52]. Multiomics integration platforms like Sangerbox 3, MOFA+, and MixOmics improve the visualization of relationships between datasets [53, 54, 55]. For single‐cell RNA‐seq analysis, tools such as Scanpy and Seurat aid in clustering and in‐depth examination of expression variations, providing insights on the cellular heterogeneity of cancer [56, 57, 58]. Additionally, platforms like pyBioPortal and cBioPortal enable the integration of transcriptomic data with clinical and genomic information, enhancing the overall understanding of cancer biology [59, 60].

For RNA‐seq quantification, Salmon and Kallisto utilize ultra‐fast, alignment‐free techniques to estimate precise transcript numbers, while STAR and HTSeq offer alignment and read counting for gene expression studies [61, 62, 63, 64]. In addition, single‐cell‐specific tools like CellBender improve data quality by filtering out ambient RNA, and deep learning models such as scVI support imputation and clustering analyses [65, 66]. Furthermore, Dyngen and scRNASeqDB have advanced single‐cell research methods which facilitate gene expression data simulation and provide dynamic platforms for cross‐cancer exploration [67, 68, 69]. Tools like scissor link single‐cell RNA‐seq data to clinical outcomes, integrating cellular insights with survival rates and treatment responses, thus paving the way for translational oncology applications [70]. As technological advancements progress, transcriptomics leads the way in precision oncology, presenting exceptional opportunities to decode cancer's molecular complexities and create targeted therapeutic interventions.

### 2.3 Proteomics

Proteomics is the comprehensive study of all proteins generated by a genome and plays a crucial role in biomedical research, notably in precision oncology [71]. Unlike genomics and transcriptomics, which infer gene expression mainly from genetic sequences, proteomics seeks to reveal the functional consequences of genes: specifically, at the protein level that affect cellular operations and define phenotypes [72]. Additionally, proteomics facilitates the direct quantification of protein levels and critical posttranslational modifications (PTMs) such as phosphorylation, glycosylation, and ubiquitination, offering valuable insights into the functional status of proteins linked to cancerous changes. Proteins discovered through proteomic analyses can provide important information about tumor characteristics, patient outlook, and possible treatment responses. For example, well‐known biomarkers such as CA‐125 and PSA are used in ovarian and prostate cancers, respectively [73, 74]. By integrating proteomic assessments into clinical workflows, healthcare providers can modify treatment plans according to a patient's molecular profile, ultimately improving treatment effectiveness and patient outcomes.

Recent advancements in mass spectrometry (MS) and protein microarray technologies have positioned proteomics at the forefront of scientific inquiry [75]. Techniques such as liquid chromatography–tandem mass spectrometry (LC–MS/MS) are widely utilized for high‐throughput protein analysis [76]. These sophisticated methods possess the capability to detect thousands of proteins within complex biological samples and quantify their abundance, thereby facilitating comprehensive tumor proteome profiling. Protein microarrays, an additional critical tool in the field of proteomics, allow for the simultaneous investigation of multiple proteins and their interactions with various molecules [77]. By presenting arrays of known proteins on a solid substrate, this technology enables high‐throughput screening of protein expression, interactions, and functions in an exceptionally efficient and informative manner.

To effectively utilize proteomics in oncology, it is essential to have robust bioinformatics tools for managing and analyzing the vast data generated by these studies. As the field advances, computational platforms that support biomarker discovery have become increasingly important. Software like MaxQuant and Skyline enables precise quantification of protein levels and PTMs, allowing for comparative analyses between tumor and normal tissues or between sensitive and resistant tumors [78, 79]. Moreover, tools like Proteome Discoverer and PeptideShaker streamline workflows for protein identification, while OpenMS provides an open‐source solution for LC–MS data analysis [80, 81, 82, 83]. For exploring protein interactions, tools such as STRING, Cytoscape, and BioGRID aid in mapping complex protein networks, and PhosphoSitePlus offers a comprehensive catalog of verified PTMs [84, 85, 86, 87]. Structural prediction software, including Alphafold and I‐TASSER, reveals insights into protein function, supported by visualization tools like Pymol [88, 89, 90]. High‐throughput tools such as MS‐DIAL and DIA‐NN, alongside ML applications like DeepNovo, improve peptide identification and sequencing precision [91, 92, 93]. Additionally, statistical analysis platforms like Perseus and limma/EdgeR in R allow for detailed interpretation of proteomic data, ensuring its biological relevance while minimizing false discoveries [94, 95].

The integration of these advanced tools facilitates a substantial enhancement in our comprehension of the molecular foundations of cancer, concurrently contributing to the development of molecular biomarkers, targeted therapies, and personalized treatment strategies.

### 2.4 Epigenomics

Cancer develops not solely from genetic mutations but also from significant epigenetic alterations that impact gene expression and cellular behaviors, including uncontrolled growth, invasion, and metastasis [96]. For instance, the hypermethylation of tumor suppressor genes may reduce their expression, whereas hypomethylation can potentially activate oncogenes, thereby contributing to malignancy. These epigenetic processes are essential for the identification of biomarkers that can inform personalized treatment strategies. Epigenomics, which investigates epigenetic modifications of genetic material, represents an emerging field in precision oncology [97]. In contrast to genomics, which focuses on DNA sequences, epigenomics examines how alterations such as DNA methylation, histone modifications, and noncoding RNA interactions influence gene expression. Importantly, epigenetic changes are often reversible, rendering them promising targets for therapeutic intervention. Currently, a variety of epigenetic modulators capable of altering these changes are undergoing evaluation in clinical trials, emphasizing the importance of epigenomics in cancer care [98].

Recent advances in high‐throughput sequencing technologies have revolutionized epigenomic research [99]. Methods like whole‐genome bisulfite sequencing (WGBS), chromatin immunoprecipitation sequencing (ChIP‐seq), and RNA‐seq are frequently used to map DNA methylation patterns, histone modifications, and interactions with noncoding RNAs [100, 101, 102, 103]. For example, WGBS provides a comprehensive perspective on DNA methylation across the genome by distinguishing between methylated and unmethylated cytosines, thereby revealing methylation patterns associated with various cancers. This technique is crucial for understanding the epigenomic landscape of tumors and for identifying methylation signatures that may serve as biomarkers. ChIP‐seq enables researchers to investigate histone modifications and transcription factor binding sites throughout the genome, offering vital insights into how these changes affect gene expression in cancer. This approach can uncover regulatory elements involved in cancer progression, helping to link epigenetic changes with clinical outcomes. Moreover, the role of noncoding RNAs, including microRNAs and long noncoding RNAs (lncRNAs), has gained significant attention in epigenomics [104]. These molecules regulate gene expression and chromatin structure, which significantly impact tumor biology [104, 105, 106, 107, 108, 109]. Moreover, RNA‐seq has furthered the profiling of noncoding RNAs in various cancers, emphasizing their potential as biomarkers or therapeutic targets [110].

Bioinformatics tools are essential for analyzing the large datasets generated by epigenomic research. Bismark is often used for DNA methylation analysis, aligning sequencing data and identifying differential methylation regions at a single‐base level [111]. MethylKit and DSS are popular for detecting methylation changes under different conditions in differential analysis [112, 113]. For ChIP‐seq and ATAC‐seq data, MACS is a favored tool for detecting peaks in chromatin accessibility, while SICER is particularly effective at revealing larger enrichment areas in histone modification studies [114, 115]. Tools like DiffReps and ChromHMM provide comprehensive analyses of chromatin states and accessibility across genomic regions, emphasizing the epigenetic patterns critical for understanding cancer biology [116, 117]. To separate meaningful biological signals from background noise, researchers frequently employ limma and EdgeR, R‐based packages that assist in identifying significant epigenetic changes between cancerous and normal tissues [118, 119].

The integration of epigenomic data with bioinformatics creates numerous opportunities for identifying biomarkers in precision oncology. Epigenetic profiling has already revealed several promising biomarkers, including specific methylation patterns linked to certain cancer types and clinical outcomes [120]. For example, the methylation status of genes such as RASSF1A and GSTP1 has been investigated as a potential biomarker for diagnosing prostate cancer [121]. Additionally, epigenomic biomarkers can help in making therapeutic choices [122]. Genes exhibiting abnormal methylation can support the selection of patients for epigenetic treatments, while biomarkers related to drug response and resistance can inform personalized treatment plans based on an individual's epigenetic profile.

### 2.5 Metabolomics

Cancer is frequently characterized by altered metabolic pathways, particularly exemplified by the Warburg effect, wherein tumor cells predominantly favor glycolysis followed by lactic acid fermentation for energy production, even in the presence of oxygen [123]. This modification in metabolic processes facilitates enhanced cellular proliferation and contributes to tumor progression and metastasis. Metabolomics, defined as the comprehensive analysis of metabolites within biological systems, yields significant insights into the biochemical processes driving cancer biology [124]. Given that metabolites represent the end products of cellular activities, they reflect the physiological conditions of cells and tissues, thereby revealing critical information regarding metabolic dysregulation in cancer. Within the field of precision oncology, where treatments are tailored to individual patient profiles, metabolomics proves indispensable for identifying biomarkers [125]. Advancements in metabolomics and bioinformatics have already discovered several promising cancer biomarkers applicable for detection and prognosis. For example, elevated levels of metabolites such as 2‐hydroxyglutarate have been correlated with specific cancers, including glioma and acute myeloid leukemia, thereby presenting new opportunities for early detection and personalized treatment [126, 127]. Furthermore, metabolomic profiling can enhance therapeutic strategies by identifying metabolites associated with drug resistance or sensitivity [128]. For instance, alterations in lipid metabolism have been linked to chemotherapy responses in breast cancer, suggesting that modifying treatments based on metabolic profiles may enhance efficacy [129].

Metabolomics has wide‐ranging clinical applications in oncology. Metabolites can serve as early diagnostic markers, enabling the identification of cancer even in asymptomatic stages [130]. Additionally, metabolic profiles provide prognostic information, assisting in identifying patients at an elevated risk of recurrence or treatment resistance. Furthermore, metabolites associated with therapeutic response can inform personalized treatment strategies, enhancing targeted therapies’ effectiveness [128]. MS and nuclear magnetic resonance (NMR) spectroscopy are the principal techniques utilized for metabolomic profiling [131, 132]. MS is extensively used due to its sensitivity and capability to analyze complex mixtures. When integrated with chromatographic techniques such as gas chromatography (GC–MS) or liquid chromatography (LC–MS), MS facilitates both qualitative and quantitative analysis of a wide array of metabolites [133, 134]. This versatility makes MS an invaluable tool for investigating tumor metabolism. NMR, while less sensitive, offers distinct advantages, including nondestructive testing and the ability to ascertain metabolite structures. It is particularly beneficial for profiling metabolites within intact biological matrices, such as tissues or biofluids, and can corroborate findings from MS‐based investigations due to its reproducibility and quantitative attributes.

The rapid expansion of metabolomics has led to vast datasets that require advanced computational and bioinformatics tools for effective analysis and interpretation. For instance, XCMS is commonly used for processing MS data, including peak detection, retention time adjustment, and metabolite quantification to uncover cancer‐specific metabolic alterations [135]. Other popular tools such as MZmine and OpenMS facilitate the analysis of LC–MS and GC–MS liquid data, along with Sangerbox 3, which supports multiomics integration [136]. Furthermore, tools like MetaboAnalyst and MZmine play a crucial role in managing raw metabolomic data, performing peak identification, retention time adjustment, and normalization, thus ensuring high‐quality data for further analyses [137, 138].

In metabolite identification, MetFrag is a valuable tool for in silico fragmentation and matching MS/MS data [139]. On the other hand, Global Natural Products Social (GNPS) provides web‐based resources tailored for sharing and analyzing MS/MS data, particularly focused on natural products research [140]. Moreover, databases like the Kyoto Encyclopedia of Genes and Genomes (KEGG), the Human Metabolome Database (HMDB), and Reactome provide crucial information about biological pathways related to metabolites, aiding in pathway enrichment analysis [141, 142]. KEGG organizes pathways by merging genomic, chemical, and functional data, in cancer biology. Additionally, specialized software tools such as LipidSearch, LIMSA, and LipidBlast significantly improve the identification and quantification of lipids, thereby expanding the focus of metabolomic studies to include lipidomics, a vital component of cancer metabolism [143].

In metabolomics, statistical tools like R packages limma and glmnet, alongside software such as SPSS and SAS, help researchers conduct multivariate analyses, including principal component analysis (PCA) and partial least squares discriminant analysis [144, 145]. These methods aid in distinguishing metabolic patterns among various cancer types or clinical subgroups, revealing potential biomarkers linked to particular tumor traits or treatment responses.

Researchers are enhancing the identification of biomarkers that can substantially improve cancer diagnosis, prognosis, and therapeutic monitoring by integrating advanced bioinformatics tools with metabolomic analyses. Consequently, metabolomics presents significant potential for the future of precision oncology, paving the way for personalized treatment strategies (Table 1).

**TABLE 1:** Current bioinformatics tools for biomarker discovery in precision oncology, sub‐categorized to omics level, function, and application limitations.

Omics	Category	Tools	Description	Limitations	References
Genomics	Gene expression analysis	DESeq2, EdgeR, limma, Cufflinks, Ballgown	Differential expression analysis of RNA‐seq and microarray data	Limited performance with extreme gene expression levels; normalization and quality control are essential	[21]
	Variant calling	GATK, MuTect2, Haplotype Caller, VarScan, FreeBayes	Tools for variant discovery, SNPs, and somatic mutation calling	GATK requires large computational resources; performance can vary depending on reference genome quality.	[146]
		Strelka, Lofreq, Platypus	High‐sensitivity variant callers	May require extensive filtering; complex workflows	[147]
	Functional genomics	GSEA, DAVID, KEGG, Reactome, Sangerbox 3	Gene set enrichment, pathway analysis, and functional annotation	May overlook context‐specific pathways or interactions, relying heavily on gene annotations that can differ across databases	[148]
	Copy number alterations	CNVkit, Control‐FREEC, ExomeCNV	Detection of copy number variations from exome or whole genome data	Sensitivity depends on sequencing depth and genome complexity, with noisy data increasing the risk of false positives.	[149]
Epigenomics	DNA methylation	Bismark, MethylKit, Bis‐SNP, DSS,	Tools for methylation calling and differential methylation analysis from bisulfite sequencing	Sensitive to sequencing errors in bisulfite‐treated reads, with difficulty accurately calling methylation status in repetitive regions	[150]
	Chromatin accessibility	MACS, SICER, ChIPseeker, ATAC‐Seq Tools (HMMRATAC)	Tools for peak calling, analysis of chromatin accessibility (e.g., ChIP‐seq, ATAC‐seq)	Difficult to distinguish between biologically relevant peaks and noise in low‐signal data; requires deep sequencing	[151]
	Epigenetic regulation	EpiDISH, MethylSig, BSmooth	Tools for deconvolution of methylation data and analysis of epigenetic regulation	Interpretation of epigenetic regulation is context dependent; there are limited databases for noncoding regions.	[152]
	Histone modification analysis	diffReps, RSEG, ChromHMM	Analysis of histone modification data from ChIP‐seq	False positive peaks and misalignment of histone marks can lead to inaccurate functional predictions.	[153]
Proteomics	Protein identification	MaxQuant, Proteome Skyline, Discoverer, MSFragger	Tools for mass spectrometry‐based protein identification and quantification	MS often misses low‐abundance proteins; identification relies on high‐quality spectral libraries.	[78]
		Trans‐proteomic pipeline, Open MS, Peptide Shaker,	Open‐source tools for peptide and protein identification	High data preprocessing burden, especially for large datasets; lower sensitivity compared with commercial tools.	[154]
	Protein–protein interaction	STRING, Cytoscape, BioGRID, IntAct	Visualization and analysis of protein interaction networks	Often relies on predicted interactions, which can result in false positives; limited experimental validation	[155]
	PTMs	PhosphoSitePlus, PTMScan, MODa	Tools for identifying and analyzing PTMs	PTM analysis is highly sensitive to sample preparation and detection techniques and has limited coverage of PTM types.	[87]
	Protein structural analysis	AlphaFold, Pymol, I‐TASSER, MODELLER	Tools for predicting and visualizing protein structures	Prediction accuracy decreases for highly disordered regions or multidomain proteins; it is limited for protein interaction predictions.	[156]
Metabolomics	Metabolite identification	XCMS, Metabo Analyst, MZmine, OpenMS, Sangerbox 3	Tools for metabolite identification and quantification from MS data	Limited spectral libraries for certain metabolite classes; metabolite annotation is challenging due to overlap in mass/charge ratio.	[157]
		MetFrag, GNPS	Fragmentation‐based metabolite identification	High false discovery rates for low‐resolution MS; limited databases for novel compounds	[158]
	Metabolic pathway analysis	KEGG, HMDB, MSEA, Reactome, Pathway Commons	Pathway and network analysis tools for connecting metabolomics data to biological functions	Pathways are often curated from general organism models, which might not reflect species‐specific or condition‐specific pathways.	[159]
	Lipidomics	LipidSearch, LIMSA, LipidBlast	Tools for analyzing lipidomics data from NMR or MS	Lipid identification is challenging due to the diversity of lipid structures; it requires specific libraries and standards.	[160]
Transcriptomics	Bulk RNA‐seq	Salmon, Kallisto, STAR, RSEM, HTSeq	RNA‐seq quantification tools for transcript‐level and gene expression analysis	Transcript quantification accuracy can drop for low‐expressed genes or those with highly similar sequences.	[161]
	Single‐cell RNA‐seq	Seurat, Scanpy, CellRanger, Monocle, Sangerbox 3	Tools for clustering, differential expression, and trajectory analysis of single‐cell RNA‐seq data	Single‐cell RNA‐seq data often has high dropout rates; clustering can be influenced by noise, making cell type annotation complex.	[162]
	Noncoding RNA Analysis	miRDeep2, Infernal, snoSeeker, lncRNAtor,	Tools for identification of noncoding RNAs (*miRNAs*, snoRNAs, *lncRNAs*) from *RNA‐seq*	Noncoding RNAs can be difficult to detect due to short lengths or sequence similarity; functional annotation is often incomplete.	[163]
Integrative multiomics	Multiomics integration	iCluster, MixOmics, MOFA+, Galaxy, Sangerbox 3	Tools for integrating genomics, proteomics, transcriptomics, and metabolomics data	The integration of multiomics datasets can be computationally intensive and requires careful interpretation of correlations between different omics layers.	[55]
Cancer‐specific tools	Cancer genomics & biomarkers	cBioPortal, GEPIA, OncoKB, TIMER, Sangerbox 3	Tools for cancer biomarker discovery and analysis of public datasets like TCGA, GTEx	Limited to public cancer datasets; may not capture rare mutations or ethnic‐specific biomarker data.	[164]
	Drug sensitivity prediction	GDSC, PRISM Repurposing, CellMiner, PharmacoDB	Tools for predicting drug sensitivity and resistance based on cancer cell line data	in vitro data may not fully reflect real‐world drug responses due to tumor microenvironment and patient variability.	[165]
	Immune infiltration analysis	TIMER, CIBERSORT, xCell, EPIC	Tools for estimating immune cell infiltration from bulk RNA‐seq and other datasets	Immune cell quantification accuracy depends on deconvolution algorithms, which may perform poorly on low‐purity or heterogeneous tumor samples.	[166]

References [61, 95, 176, 177, 178, 179, 180, 181, 182, 183, 184, 185, 186, 187, 188, 189, 190, 191, 192, 193, 194, 195, 196, 197] present evidence regarding the applications and limitations of the listed tools across different domains. Genomics tools, including those for differential expression analysis and variant calling, encounter challenges such as performance variability in extreme expression values and high demands for computational resources. Functional genomics tools, especially those employed for pathway enrichment analysis, might face issues tied to pathway relevance, as their precision relies on the quality and completeness of gene annotations. Epigenomics tools, which analyze DNA methylation and chromatin accessibility, often find it difficult to accurately detect methylation in repetitive regions and to differentiate significant peaks. Tools for proteomics and metabolomics aid in the identification of proteins and metabolites; nonetheless, their sensitivity tends to be limited when it comes to detecting low‐abundance analytes. Transcriptomics tools, whether used for single‐cell or bulk RNA sequencing, experience challenges from noise and dropout events affecting lowly expressed genes, complicating cell type annotation. Multiomics integration tools, vital for thorough analyses, necessitate careful interpretation of data due to the complexities involved in merging diverse data types. Cancer‐specific tools, frequently utilized in biomarker discovery, estimation of immune infiltration, and prediction of drug sensitivity, primarily depend on public datasets. Consequently, their accuracy may fluctuate, especially in heterogeneous tissue environments or when examining rare mutations.

## 3 Key Bioinformatics Tools for Biomarker Discovery

**FIGURE 2:**
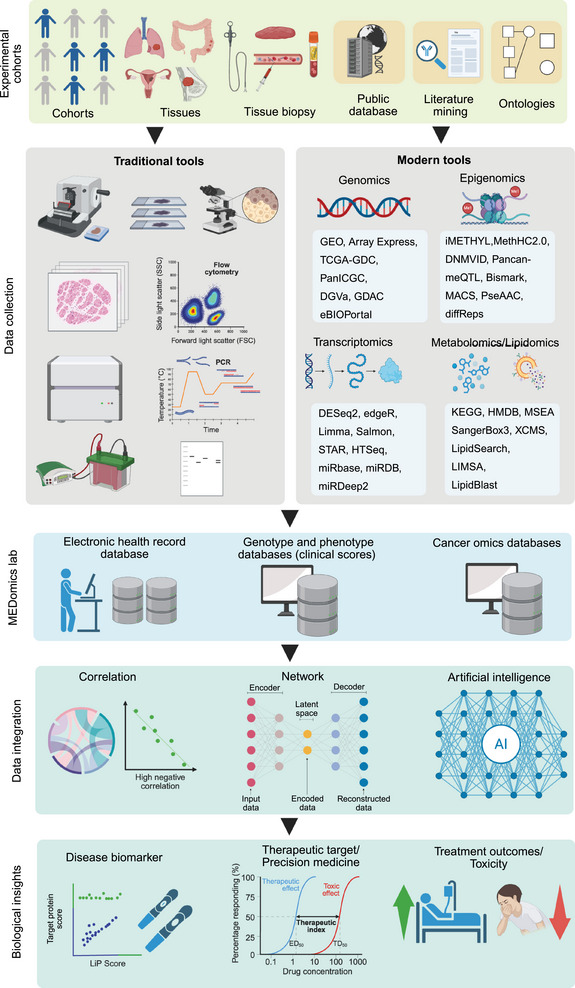
Detailed workflow showing the combination of in silico and wet lab methods for generating biological insights and developing oncotherapeutic strategies in precision medicine: This diagram depicts the smooth integration of computational (in silico) and experimental (wet lab) techniques to extract biological insights and formulate oncotherapeutic strategies for precision medicine. The process initiates with gathering data from various sources, including clinical cohorts, patient‐derived tissue samples, biopsies, publicly accessible databases, literature reviews, and ontologies that guarantee standardized knowledge representation. Standard laboratory methods like histopathology, flow cytometry, gel electrophoresis, and spectrophotometry are enhanced by high‐throughput omics technologies, encompassing genomics, epigenomics, transcriptomics, and metabolomics/lipidomics. These data are meticulously organized and analyzed through specialized bioinformatics tools and repositories, such as GEO, TCGA, and HMDB. These datasets are integrated in MEDomics labs, leveraging electronic health records, genotype–phenotype connections, and cancer‐specific multiomics repositories for thorough data harmonization. Subsequently, computational analyses are utilized to draw significant insights—correlation studies disclose biological relationships, network modeling portrays the complexity of molecular interactions, and AI methodologies, including machine learning and deep learning, assist in pattern recognition, predictive modeling, and simulation of biological processes. The outcome of these combined efforts leads to actionable oncotherapeutic strategies, such as uncovering disease‐specific biomarkers, identifying new therapeutic targets, developing personalized medicine approaches, and assessing treatment efficacy and potential toxicity. This comprehensive framework promotes a data‐driven and mechanistic understanding of cancer biology, thereby propelling precision oncology through advanced bioinformatics and experimental strategies. The figure is generated using BioRender.com. GEO: Gene Expression Omnibus, ArrayExpress: a public database for functional genomics data, TCGA: The Cancer Genome Atlas, DNAm: DNA methylation, cBioPortal: a tool for bioinformatics and genomics data exploration, DESeq2: differential expression analysis based on *RNA‐seq* data, edgeR: a bioconductor package for differential gene expression analysis, limma: linear models for microarray data, Salmon: *RNA‐seq* quantification tool, STAR: spliced transcripts alignment to a reference (*RNA‐seq* aligner), HTSeq: a Python package for *RNA‐seq* data processing, miRBase: a database for microRNA sequences, miRDDB: a database for microRNA‐target interactions, miRDeep2: a tool for identifying novel microRNAs, MethyC‐2.0: a methylation data analysis tool, DNMT3: DNA methyltransferase 3D, Pancan‐mQTL: pan‐cancer methylation Quantitative Trait Loci, Bismark: a tool for mapping bisulfite‐treated sequencing data, MiSeq: a sequencing platform by Illumina, KEGG: Kyoto Encyclopedia of Genes and Genomes, HMDB: Human Metabolome Database, MSEA: metabolic set enrichment analysis, SangerBox3: a software for genomics and bioinformatics analysis, XCMS: a tool for processing liquid chromatography–mass spectrometry (LC–MS) data, LipidBlast: a comprehensive lipid mass spectrometry database, LIP score: a score used for assessing lipidomics‐based biomarkers.

The development of bioinformatics tools has revolutionized biomarker discovery in precision oncology, enabling researchers to conduct a comprehensive analysis of complex datasets. Identifying biomarkers in precision oncology requires employing specialized bioinformatics tools throughout various phases of data analysis, from preprocessing to validation [167] (Figure 2). Each stage is essential to confirm that the identified biomarkers are reliable, relevant, and clinically significant. Below is an in‐depth discussion of the primary bioinformatics tools utilized for biomarker discovery.

### 3.1 Data Preprocessing and Quality Control Tools

Data preprocessing and quality control are essential steps in analyzing high‐throughput sequencing data, enhancing the integrity and reliability of subsequent analyses. The process typically includes several steps, such as trimming low‐quality reads, removing sequencing adapters, and filtering out contaminants. This initial step is crucial for improving the integrity and reliability of subsequent analyses [168].

The quality control of sequencing data performed during the preprocessing phase. FastQC is a widely utilized tool for assessing the quality of raw sequencing data, generating comprehensive reports on base quality scores, GC content, and sequencing depth. Researchers utilize these reports to identify potential data issues, such as low‐quality reads or biases, which may adversely affect the analysis [169].

Upon assessing the quality, Trimmomatic is used to trim low‐quality bases and eliminate sequencing adapters, thereby ensuring the retention of only high‐quality reads for subsequent analyses [170]. This procedure significantly enhanced the accuracy of variant calling and gene expression quantification, establishing a robust foundation for data interpretation. Following preprocessing, normalization is often essential to address variations attributable to discrepancies in sequencing depth or batch effects. ComBat and ComBat‐ref are frequently employed to adjust for batch effects arising from technical variations in sample preparation or sequencing, thereby preventing false biomarker identification [171]. Furthermore, surrogate variable analysis (SVA) serves to detect and remove batch effects and other unwanted variations that are not directly measurable [172]. SVA is widely applied in RNA‐seq and microarray studies to uphold data comparability across samples and experiments, ultimately improving the reliability of analytical results [173].

### 3.2 Biomarker Discovery Algorithms

Once the quality of data is assured, the subsequent step involves identifying potential biomarkers through computational algorithms that differentiate between relevant features (such as genes or proteins) and background noise. Feature selection methods, including Lasso and Elastic Net, are employed to mitigate the complexities of large datasets and to select the most predictive biomarkers [174]. Lasso is particularly advantageous for high‐dimensional genomic data, while Elastic Net is adept at handling correlated datasets with interdependent variables [175, 176]. ML methods play a crucial role in biomarker discovery by uncovering intricate relationships within extensive datasets [177]. Random Forests amalgamate decision trees to facilitate robust feature selection, whereas support vector machines (SVMs) are utilized to differentiate between cancerous and noncancerous samples [178, 179]. Furthermore, deep learning techniques, especially in the context of multiomics and image data, unveil complex patterns through the application of neural networks [180].

The primary instruments used in ML include Random Forest and scikit‐learn, with a focus on model training in R, along with TensorFlow and PyTorch for deep learning applications [181, 182, 183, 184, 185]. Furthermore, additional tools such as Weka, Bioinformatics Toolbox, and R/Bioconductor offer methodologies for statistical analysis [186, 187]. In addition, Cytoscape and GenePattern facilitate the visualization of biomarker interactions within pathways, thereby assisting in the interpretation of biomarker networks in the context of precision oncology [188, 189].

### 3.3 Multiomics Integration Platforms

Biomarkers are rarely identified through the utilization of a singular data type, as cancer encompasses intricate interactions across molecular strata. Multiomics integration platforms connect genomic, transcriptomic, proteomic, epigenomic, and metabolomic data, offering a holistic perspective on cancer biology and robust biomarkers supported by cross‐omics evidence. Tools to integrate the multiomics data include SNF and *iCluster*. SNF constructs similarity networks for each omics category, which are then merged into a consensus network [190, 191]. This process facilitates biomarker discovery with consistent evidence across data layers. In comparison, *iCluster* groups multiomics data to identify cancer subtypes through common molecular traits, which may reveal biomarkers specific to subtypes that are relevant for prognosis or treatment response [192, 193].

Network‐based methods are essential for identifying biomarkers. Tools like netboxr combine protein–protein interaction networks with genomic information, pinpointing regulatory pathways and biomarkers across various omics layers [194]. Cytoscape acts as a flexible platform to visualize molecular interaction networks, integrate multiomics data, and investigate biomarkers within biological pathways and network frameworks [188]. Moreover, Cytoscape serves as a platform for visualizing these networks and performing pathway enrichment analyses through various plugins, such as ClueGO and Bingo These tools enhance the biomarker discovery domain and encourage personalized strategies in oncology (Figure 2).

### 3.4 Pathway and Network Analysis

In cancer biomarker discovery, there is a growing emphasis on analyzing pathways and networks rather than solely examining individual genes or proteins. This transition recognizes the connections among molecular processes. Disruptions in biological pathways can occur due to altered gene expression or protein activity, and recognizing these changes may facilitate the development of robust biomarkers.

A range of tools enhances pathway enrichment and network development. Gene set enrichment analysis (GSEA) detects enriched gene sets or pathways crucial for understanding differential gene expression in biological contexts [195]. The database for annotation, visualization, and integrated discovery (DAVID) aids in functional annotation, highlighting enriched pathways and cellular components [196, 197]. The Reactome provides a curated collection of biological pathways and mapping tools that relate data to pathway disruption for biomarker identification [198, 199]. Ingenuity pathway analysis (IPA) is utilized for pathway analysis and network construction, widely applied in therapeutic biomarker research [200]. Network analysis helps identify biomarkers by examining genes or proteins within networks, focusing on key “hubs” that regulate vital processes. STRING creates protein–protein interaction networks and identifies interactions specific to cancer [155]. Another essential bioinformatics tool known as BioGRID used to display essential protein and genetic interactions for networks in cancer biology [201]. *ClusterProfiler*, an R package, enhances these tools by visualizing pathway enrichment and aiding in dataset interpretation [202].

Recently developed tools have significantly advanced the discovery of biomarkers using network‐based approaches. For instance, NetPath meticulously curates the signaling pathways associated with cancer, facilitating the identification of biomarkers through the dysregulation of these pathways [203]. Metascape, on the other hand, consolidates various databases for pathway enrichment and network analysis, while GeneMANIA predicts gene functionality based on coexpression and interaction networks [204, 205]. Collectively, these tools empower researchers to identify biomarkers by systematically analyzing critical network nodes and dysregulated pathways.

### 3.5 Validation of Biomarkers

Validating biomarkers is a complex yet crucial process for translating research findings into clinical trials and applications [206]. Biomarker candidates that have successfully completed the initial verification phase and developed precise, robust quantitative assays must then undergo in silico, analytical, and clinical validation to ensure reliability and clinical relevance. In silico validation, which encompasses computational evaluations, has emerged as an integral part of the validation process for potential biomarkers [207]. This method employs mathematical modeling, simulations, and data analysis from publicly accessible datasets such as TCGA and Gene Expression Omnibus (GEO). These resources offer comprehensive multiomics and gene expression data from thousands of patients across a variety of cancer types. Specifically, TCGA plays a pivotal role in validating genetic and RNA‐based biomarkers, while GEO emphasizes RNA‐based biomarkers in a range of clinical conditions and cancers [208, 209]. Supplementary databases like ArrayExpress and the International Cancer Genome Consortium (ICGC) provide functional genomics validation through diverse experimental designs, allowing for the assessment of somatic mutations CNVs [210, 211]. Additionally, meta‐analytic strategies enhance the validation process by combining data from numerous studies, thereby increasing statistical power and confirming the robustness of potential biomarkers [212, 213].

Bioinformatics tools offer numerous benefits, such as cost efficiency, comprehensive evaluations of potential biomarkers, and improved survival analyses alongside risk assessments in the validation process. Survival analyses, including Kaplan–Meier (KM) curve analyses utilizing TCGA and GEO datasets, allow researchers to link biomarker expression with prognostic results, including overall survival, progression‐free survival, and disease‐free intervals [214]. SurvExpress, an extensive gene expression database and online biomarker validation resource, enhances risk stratification by combining survival analysis with gene expression profiles, confirming the predictive value of biomarkers in separate cohorts [215]. For sophisticated statistical modeling, R‐based packages like Survminer utilized to generate KM curves and Cox proportional hazards (Coxph) models [216]. The Coxph model is vital for examining the relationships between survival time and their predictors [217]. Web platforms such as GEPIA connect TCGA and GTEx data for gene expression and survival examination, investigating the prognostic significance of particular genes [218]. Additionally, tools like TIMER extend biomarker validation by factoring in immune cell infiltration dynamics with survival information, aiding in the assessment of how immune cells and gene expression affect survival rates [219]. Additionally, specialized bioinformatics platforms like OncoLnc and Sangerbox 3 streamline survival analyses utilizing TCGA‐derived molecular profiles (e.g., mRNA, miRNA, lncRNA) to investigate survival outcomes [220]. Prognoscan offers meta‐analysis capabilities to assess the connections between survival and gene expression, while the TCGA Biolinks (R package) facilitates large‐scale survival research by maintaining reproducible access and analysis of TCGA data, thereby preserving methodological rigor in biomarker validation [221].

One essential aspect of bioinformatics tools involves performing biomarker analyses after an in vivo or in vitro efficacy study, which assists in validation efforts by correlating targets with the drug's mechanism of action [222]. This process helps predict the response or nonresponse of potential biomarkers. The findings from these analyses can serve as a basis for establishing inclusion and exclusion criteria for future clinical studies and validations. Bioinformatics tools systematically analyze downstream data from extensive in vitro assays, incorporating cell lines and genomically annotated tumor organoids, enabling researchers to differentiate between responding and nonresponding cell populations based on unique genomic signatures, thus providing early indications of possible biomarkers. By merging pharmacological response data from these screenings with computational models, researchers can link drug sensitivity or resistance with various genomic features such as gene expression profiles, mutational landscapes, CNVs, and pathway activation states, to formulate robust, multidimensional biomarker hypotheses. Promising candidates identified in vitro can progress to in vivo validation in preclinical models, where functional and mechanistic studies sharpen their biological significance [222]. Bioinformatics‐based approaches play a crucial role in choosing xenograft or *patient‐derived xenograft* models that align with a drug's molecular target or mutation profile, thereby ensuring translational relevance. This iterative process links in silico predictions to experimental validation, assisting in the prioritization and confirmation of the most clinically significant biomarkers potential [223].

Advanced analytical validation models, including AI and ML, enhance the validation process through cross‐validation, which rigorously assesses the application of biomarkers across various datasets [224]. This enables evidence‐based decisions about their clinical usefulness. Such an approach prevents overfitting, where a model too closely aligns with training data, impairing its ability to generalize to new data. Effective analytical validation assures both accuracy and generality of the model. Clinical validation is a crucial aspect of biomarker validation, as it improves the reliability of results and verifies the clinical significance of potential biomarkers by assessing their sensitivity and specificity [225]. Additionally, clinical validation clarifies the relationship between a biomarker and clinical aspects like treatment response, disease stage, and comorbid conditions. This validation relies on both in silico and analytical methods, which may involve retrospective reviews of past clinical trial data or new prospective trials. Retrospective reviews serve as a form of external clinical validation, particularly when biomarker assessments were not included in the original study design. In contrast, prospective clinical trials showcase the clinical relevance of a biomarker, acting as a kind of external validation that illustrates how its application can improve health results [226]. Various prospective clinical trial designs aim to confirm the clinical utility of biomarkers. A significant case is the United States Food and Drug Administration (US FDA's) 2017 tissue‐agnostic approval of pembrolizumab, the first treatment authorized based on a biomarker rather than tumor location [227]. This decision stemmed from the KEYNOTE‐016 study, which indicated higher overall response rates in patients with microsatellite instability‐high (MSI‐H) tumors treated with pembrolizumab compared with those with microsatellite stable (MSS) tumors, regardless of cancer type. Regulatory approval was based on pooled results from five trials (total *N* = 149), with MSI‐H status retrospectively identified in 14 patients from two prospective studies, while 135 patients from three additional trials were prospectively confirmed. The objective response rate for MSI‐H patients was 39.6% (including a 7% complete response rate) across 15 tumor types, which is deemed clinically significant. In contrast, patients with MSS tumors in the KEYNOTE‐016 trial had a 0% response rate, underscoring the predictive power of the biomarker [228]. Another significant prospective clinical trial is the EURTAC trial, which resulted in the US FDA approval of erlotinib as a first‐line therapy for metastatic NSCLC with EGFR mutations [229]. Other examples of such trials include the MARVEL trial and SWOG S0819 [230, 231].

## 4 AI and ML in Biomarker Discovery

AI and ML have become pivotal technologies in oncology, especially for identifying predictive biomarkers in precision medicine [232, 233, 234, 235, 236, 237, 238]. By facilitating the examination of extensive and intricate datasets, AI methods offer robust tools for discovering new biomarkers that aid in diagnosis, prognosis, and treatment choice. This section explores the role of AI and ML in biomarker discovery, emphasizing their methodologies, resources, and potential for the future (Table 2).

**TABLE 2:** AI/ML approaches in cancer biomarker discovery.

Approach	Application	Emerging trends	Practical challenges	Tools	Notable studies	References
DL	Analyzing complex omics and imaging data to identify biomarkers	CNNs for genomic sequence analysis, RNNs for time‐series data analysis	Data sparsity, overfitting on small datasets	TensorFlow, Keras, Caffe, PyTorch	Immune gene signatures in ovarian cancer, leveraging DL models	[18]
SL	Predicting cancer subtypes or patient outcomes based on biomarkers	Support vector machines, random forests, gradient boosting	Need for large labeled datasets, class imbalance	Scikit‐learn, XGBoost, LightGBM	Seven‐gene signature for lung cancer prognosis using supervised ML	[239]
UL	Clustering patients based on omics data, identifying novel cancer subtypes	Dimensionality reduction techniques, clustering algorithms like K‐means	Lack of labeled data, interpretation of clusters	PCA, t‐SNE, UMAP, k‐Means	Clustering BC subtype based on multiomics integration using UL	[240]
FL	Enabling model training across decentralized data sources, preserving privacy	Distributed learning, model aggregation without data transfer	Ensuring privacy and security across datasets	TensorFlow Federated, PySyft	Federated learning‐based cancer survival prediction method with privacy protection	[241]
TL	Leveraging pretrained models on smaller datasets to improve biomarker prediction	Fine‐tuning models on new datasets for improved accuracy	Requires pretrained models, domain adaptation	Keras, PyTorch	Deep learning for electronic cancer record data with transfer learning	[242]
XAI	Making AI predictions transparent for clinical applications	Feature importance, rule extraction, local explanation models	Lack of trust from clinicians, need for clinical validation	SHAP, LIME	LIME for explaining ML models in healthcare	[243]
MOI	Identifying multiomics signatures for comprehensive biomarker discovery	Integration of genomic, transcriptomic, and proteomic data	Data integration challenges, noise in multiomics data	iClusterPlus, OmicLearn, MOFA, MultiOmics	Integrating multiomics data for precision oncology biomarker discovery	[244]
RL	Optimizing treatment strategies based on patient responses over time	Real‐time treatment adaptation, dynamic learning from outcomes	Requires patient‐specific long‐term data, ethical concerns	OpenAI Gym, TensorFlow Agents	Personalized cancer treatment strategies tailor treatments on the basis of a patient's health status, cancer type, and stage	[245]
EL	Combining multiple models for improved biomarker prediction accuracy	Increased focus on ensemble methods for robust predictions	Complexity in model integration, computational load	RF, Gradient Boosting, Stacking	Ensemble learning for cancer biomarker validation	[246]
NLP	Mining scientific literature to identify potential biomarkers	Text mining for the extraction of novel biomarkers from unstructured data	Ambiguity in language, challenges in processing large volumes of text	SpaCy, BERT, SciSpacy	NLP for cancer biomarker discovery through literature mining	[247]
GA	Feature selection and optimization in biomarker discovery	Evolutionary algorithms to discover optimized biomarker sets	Convergence to local minima, complexity in tuning parameters	DEAP, GAlib	Genetic algorithm‐based biomarker selection for cancer diagnosis	[248]
SVM	Classification of biomarker profiles for disease prediction	Use of kernel methods for nonlinear relationships in biomarker data	Difficulty in scaling to large datasets, computational cost	LIBSVM, Scikit‐learn	Biomarker discovery using SVM for cancer prediction	[249]
GNN	Identifying biomarker relationships and gene interactions	Modeling biomarker interactions and pathways using graph structures	Lack of sufficient graph data, scalability	DGL, PyTorch Geometric	Graph neural networks for biomarker discovery in cancer	[250]
RF	Identifying relevant features for biomarker prediction	Leveraging feature importance scores for biomarker prioritization	Difficulty in interpreting large ensemble models	Scikit‐learn, R	Random forest for biomarker selection in cancer diagnosis	[251]
BN	Modeling probabilistic relationships between biomarkers	Dynamic modeling of biomarkers with uncertainty quantification	Computational complexity, need for high‐quality data	PyMC3, Netica	Bayesian networks for cancer biomarker discovery	[252]
AutoML	Automating the selection of ML models for biomarker prediction	Deployment of automated platforms for personalized biomarker discovery	Limited flexibility, over‐reliance on automated processes	Google AutoML, H2O.ai, Auto‐sklearn	AutoML for biomarker discovery in cancer	[253]
CA	Unsupervised classification of biomarkers across different conditions	Integration of clustering with single‐cell RNA‐seq data	Difficulty in interpreting clustering results, computational expense	k‐Means, DBSCAN, Hierarchical Clustering	Clustering biomarkers for cancer subtyping	[254]
DR	Reducing high‐dimensional omics data for biomarker identification	Multilevel dimensionality reduction methods to handle large omics datasets	Loss of information, difficulty in balancing accuracy and dimensionality	PCA, t‐SNE, UMAP	PCA for dimensionality reduction in biomarker discovery	[255]

Artificial intelligence (AI) and machine learning (ML) algorithms, such as deep learning (DL), supervised learning (SL), unsupervised learning (UL), and federated learning (FL), have greatly enhanced cancer research by facilitating the analysis of intricate, high‐dimensional omics data. Methods including random forests (RF), support vector machines (SVM), and graph neural networks (GNN) are particularly effective at uncovering hidden biomarkers for cancer diagnosis, prognosis, and treatment responses, outperforming conventional statistical techniques. Multiomics integration (MOI) and explainable AI (XAI) are vital for improving data interpretation and model transparency, effectively addressing black‐box decision‐making apprehensions. Transfer learning (TL) and reinforcement learning (RL) also contribute to refining predictive models by utilizing prior knowledge and adaptive learning methods. Nonetheless, substantial challenges remain, such as issues with data integration, model interpretability, and the ability to generalize across various cancer subtypes. The intricate nature of multiomics datasets, alongside the need for extensive, well‐annotated training data, highlights the necessity for enhanced data quality, algorithm transparency, and computational efficiency. Approaches like AutoML and ensemble learning (EL) are under consideration to simplify model selection and hyperparameter optimization, while genetic algorithms (GA) and Bayesian networks (BN) provide promising solutions for feature selection and probabilistic modeling. Furthermore, dimensionality reduction (DR) and clustering algorithms (CA) are crucial for preprocessing large datasets, ensuring that meaningful biological insights can be drawn. As AI/ML advances, overcoming these challenges will be essential to fully leverage its potential in precision oncology.

### 4.1 Overview of AI Techniques in Oncology

AI techniques in biomedical research are categorized into supervised and unsupervised learning methods, both effective for revealing patterns in intricate datasets [256, 257, 258]. Supervised learning involves training algorithms using labeled data with known outcomes, which helps models understand the links between inputs (like gene expression profiles) and outputs (such as cancer subtypes). Common approaches include decision trees, random forests, SVMs, and neural networks, which are employed in cancer classification and in forecasting patient outcomes based on biomarker information. In contrast, unsupervised learning tackles unlabeled data to discover hidden patterns or clusters. Techniques like clustering (e.g., k‐means and hierarchical clustering) and dimensionality reduction (e.g., PCA and t‐SNE) are instrumental in biomarker discovery, identifying patient subgroups, and simplifying data complexity [181].

Deep learning, a branch of ML, plays a crucial role in biomarker discovery by seamlessly extracting hierarchical features from raw data [259]. CNNs and RNNs excel in the analysis of genomic, transcriptomic, and imaging data. These models have proven effective for classifying tumors based on histopathological images and predicting gene mutations from sequencing data, providing powerful tools for biomarker identification and large‐scale exploration of biological patterns [260, 261].

### 4.2 AI Tools for Predictive Biomarkers

**FIGURE 3:**
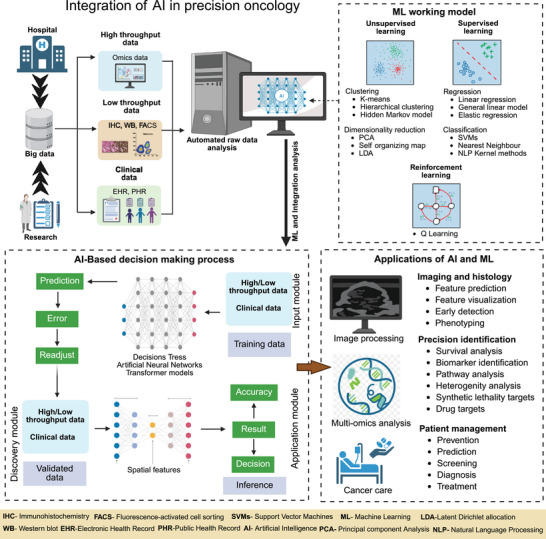
An overview of integrating artificial intelligence (AI) and machine learning (ML) into precision oncology, highlighting AI's pivotal role in cancer research and patient care. This figure depicts a comprehensive approach to precision medicine that integrates various data sources for deep phenotyping. Data collection includes public annotation databases alongside patient‐specific information, which encompasses genomic, proteomic, microbiome, clinical, epigenomic, and metabolomic datasets. Additionally, high‐throughput omics data are complemented by low‐throughput data from methods like IHC and FACS, along with clinical data sourced from EHR and PHR. The analysis of this data is powered by various machine learning algorithms, which fall into three main learning categories: unsupervised learning (e.g., clustering methods such as K‐Means and hierarchical clustering, along with dimensionality reduction techniques like PCA and LDA, supervised learning (e.g., regression models and classification algorithms, including SVMs and nearest neighbor techniques), and reinforcement learning (e.g., Q‐learning). The AI‐driven decision‐making process is illustrated as an ongoing cycle involving prediction, error analysis, and adjustments, thus improving model accuracy through the incorporation of high‐throughput and validated clinical data. As highlighted in the figure, the key applications of AI and ML in oncology include image processing, multiomics analysis, early cancer detection, phenotype characterization, biomarker discovery, pathway analysis, and holistic patient management encompassing prevention, prediction, screening, diagnosis, and treatment. The figure highlights the transformative potential of AI‐powered tools, such as decision trees, artificial neural networks, and transformer models, in enhancing precision oncology through personalized treatment strategies and improving cancer care outcomes. The figure is generated using BioRender.com. IHC: Immunohistochemistry, FACS: fluorescence‐activated cell sorting, SVMs: support vector machines, ML: machine learning, PCA: principal component analysis, NLP: natural language processing, EHR: electronic health record, PHR: public health record, AI: artificial intelligence, WB: Western blot. LDA: linear discriminant analysis, KMeans: K‐means clustering, ANNs: artificial neural networks, KNN: K‐nearest neighbors.

AI‐powered tools for biomarker discovery and predictive modeling have expedited oncology research [262]. Predictive modeling utilizes ML techniques to examine the connections between molecular traits and clinical results, including survival rates and therapeutic responses. A prime example is DeepVariant, a deep learning tool created by Google, which identifies genetic variants from NGS data and enhances mutation detection in cancer genomics [263]. PandOmics, a cloud‐based software platform that applies AI and bioinformatics approaches to multimodal omics data for biomarker discovery [264]. These tools are capable of predicting prospective biomarkers from a range of omics data (Figure 3).

Numerous specialized platforms have highlighted the critical role of AI in identifying biomarkers. OncoKB, a precision oncology knowledge base, merges genomic sequencing with clinical trial data to offer insights into gene mutations vital for cancer treatment [164]. Open‐source tools like DeepLearning4J and Omics Data Science offer deep learning libraries for complex biomarker analysis [265]. Drug Discovery AI predicts effective drug combinations based on tumor biomarkers, thereby promoting progress in personalized oncology treatments.

### 4.3 Challenges and Opportunities

AI technologies are significantly impacting biomarker discovery in oncology, yet challenges remain [266]. A key obstacle is the scarcity of high‐quality labeled data, particularly for rare cancers or newly identified biomarkers. Training effective AI models with small datasets can lead to overfitting, reducing both generalizability and clinical relevance. Although strategies like data augmentation, transfer learning, and cross‐validation can help address these problems, the lack of data continues to restrict AI's potential in biomarker discovery. Another significant challenge involves integrating various data types, such as genomics, transcriptomics, proteomics, and clinical records, which present different formats and noise levels, thereby complicating model training and efficiency [267]. Advancements in data integration techniques are vital to boost AI's capability in identifying clinically meaningful biomarkers.

Despite facing challenges, AI demonstrates significant promise in precision oncology. It facilitates the integration of diverse datasets, uncovering complex biomarker signatures that consider molecular interactions. This could lead to more accurate biomarker identification, reducing the time and costs associated with discovering predictive markers, and enabling the application of findings in clinical practice. However, the “black box” characteristic of AI models raises interpretability concerns, making clinicians reluctant to adopt these technologies [268]. Exploring explainable AI and visualization techniques is essential for nurturing clinical trust. Additionally, leveraging patient data for AI applications introduces ethical and regulatory dilemmas, necessitating well‐defined guidelines to uphold ethical integrity [269]. Collaborative efforts among researchers, clinicians, and data scientists can help tackle existing challenges, while advancements in AI technologies, like natural language processing and advanced imaging, offer fresh possibilities for biomarker discovery and therapeutic target identification [270].

## 5 Public Databases and Resources for Biomarker Discovery

**FIGURE 4:**
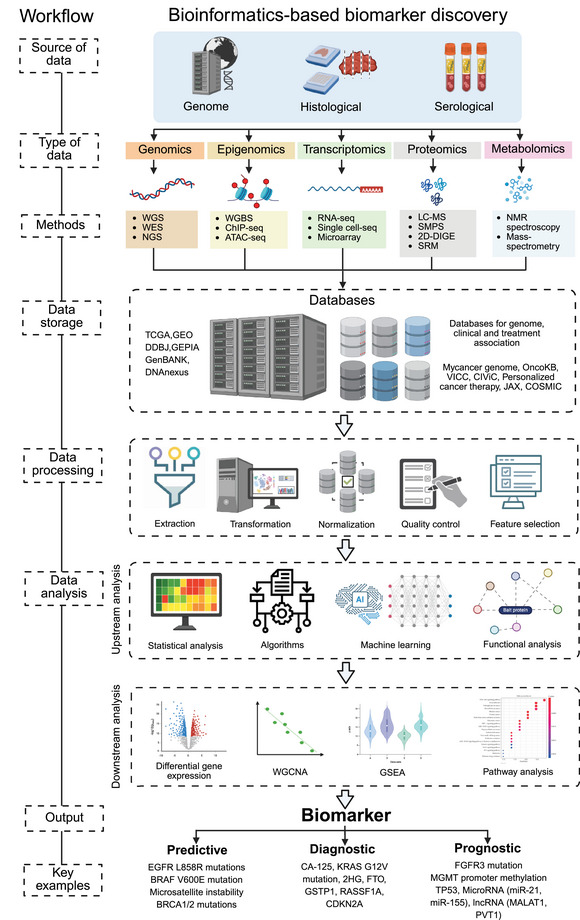
Bioinformatics‐driven workflow for biomarker discovery and validation in precision oncology. This schematic representation outlines a comprehensive bioinformatics‐driven workflow for biomarker discovery and validation in precision oncology, emphasizing the integration of diverse data sources and multiomics platforms. The workflow begins with the collection of various data types, including genomic, histopathological, and serological information derived from high‐throughput datasets and patient‐derived tissue and blood samples. These datasets incorporate multiomics approaches—genomics, transcriptomics, proteomics, and metabolomics—to identify key biomolecules such as DNA, RNA, proteins, and metabolites associated with cancer progression. Advanced technologies, including whole‐genome sequencing (WGS), RNA sequencing (RNA‐seq), whole‐exome sequencing (WES), whole‐genome bisulfite sequencing, chromatin immunoprecipitation sequencing (ChIP‐seq), assay for transposase‐accessible chromatin sequencing (ATAC‐seq), liquid chromatography–tandem mass spectrometry (LC–MS), single‐molecule protein sequencing (SMPS), two‐dimensional difference gel electrophoresis (2D‐DIGE), selected reaction monitoring (SRM), and nuclear magnetic resonance (NMR), facilitate deep phenotyping and high‐resolution molecular characterization. The data generated are systematically stored and retrieved from key repositories such as The Cancer Genome Atlas (TCGA), Gene Expression Omnibus (GEO), GenBank, DNA Data Bank of Japan (DDBJ), Gene Expression Profiling Interactive Analysis (GEPIA), Variant Interpretation for Cancer Consortium (VICC), Clinical Interpretation of Variants in Cancer (CIViC), and the Catalogue of Somatic Mutations in Cancer (COSMIC). Following data acquisition, rigorous preprocessing steps—including data extraction, transformation, normalization, and quality control—ensure reliability and reproducibility. Subsequent computational analyses employ sophisticated statistical methods, machine learning algorithms, and functional annotation techniques to derive biologically meaningful insights. Key downstream analyses, such as differential gene expression analysis, weighted gene coexpression network analysis (WGCNA), and gene set enrichment analysis (GSEA), enable the identification of putative biomarkers with potential clinical utility. The outputs of this workflow encompass predictive, diagnostic, and prognostic biomarkers, reinforcing the crucial role of bioinformatics in advancing personalized medicine and precision oncology. Identified biomarkers, including EGFR (epidermal growth factor receptor), BRCA (breast cancer gene), CA‐125 (cancer antigen 125), 2HG (2‐hydroxyglutarate), FTO (fat mass and obesity associated gene), GSTP1 (glutathione S‐transferase P), RASSF1A (Ras association domain family member 1), CDKN2A (cyclin‐dependent kinase inhibitor 2A), and FGFR3 (fibroblast growth factor receptor 3), undergo rigorous validation through multiomics integration and bioinformatics pipelines. This process ensures the identification of high‐quality biomarkers for improved cancer diagnostics, prognostic assessments, and the development of targeted treatment strategies, ultimately driving advancements in precision medicine. The figure is generated using BioRender.com.

Public databases and online resources play a crucial role in modern oncology research by providing vast datasets that help identify biomarkers for precision oncology [262]. These databases contain high‐dimensional data from various omics studies, such as genomics, transcriptomics, proteomics, and metabolomics, enabling researchers to discover potential biomarkers across multiple cancer types. By granting access to diverse datasets, public resources empower scientists to explore genetic, transcriptomic, and proteomic differences, which are essential for identifying biomarkers needed for diagnosis, prognosis, and targeted therapies (Figure 4).

### 5.1 Cancer Genomic Databases

Cancer genomic databases such as TCGA, ICGC, COSMIC, Genomic Data Commons (GDC), and cBioPortal are essential for examining genetic mutations (CNVs), and variations in gene expression associated with cancer [271]. TCGA is notable for its vast repository, which includes data from thousands of patients with various tumor types, featuring both genomic sequences and transcriptomic profiles [272]. ICGC focuses on diverse populations, aiding in the identification of genetic alterations relevant to specific demographics [273]. COSMIC specializes in somatic mutations, offering insights into actionable mutations and predictive capabilities [274]. Platforms like GDC and cBioPortal facilitate integrative analyses by combining data from numerous cancer studies and enhancing the connections between genetic alterations and clinical outcomes [274].

Additional resources will significantly enhance biomarker research beyond just genome‐level changes. The Cancer Mutation Census gathers information on mutations in genes related to cancer, facilitating the identification of common mutations [275]. Although not cancer‐specific, ExAC offers data on rare variants, contributing to a better understanding of cancer susceptibility [276]. The GEO contains extensive high‐throughput gene expression data, including essential cancer studies that are vital for comparing expression patterns in cancerous and normal tissues [277]. Together, these resources create a strong foundation for biomarker discovery, encouraging further investigation into molecular variations in cancer.

### 5.2 Transcriptomic Databases

Transcriptomic databases are essential for providing insights into gene expression, allowing researchers to probe gene regulation and identify DEGs that serve as biomarkers. GEO is a key public repository that offers high‐throughput gene expression data from various genomic techniques, such as microarray and RNA‐seq. It supports investigations into differential expression related to different cancer types, treatment responses, and disease stages. Similarly, ArrayExpress provides curated datasets from microarray and sequencing studies, enabling researchers to explore transcriptional changes linked to specific cancer phenotypes [278]. These transcriptomic resources are vital for precision oncology, assisting identification of critical biomarkers for diagnosis, prognosis, and treatment responses [278].

### 5.3 Proteomics and Metabolomics Databases

Proteomics and metabolomics databases provide useful insights into protein expression, PTMs, and metabolite profiles, facilitating the identification of biomarkers at both protein and metabolite levels. The PRoteomics IDEntifications database serves as a vital resource for MS‐based proteomics, offering essential data on protein expression, identification, and modifications that are crucial for discovering oncology biomarkers [279]. Furthermore, PeptideAtlas compiles high‐confidence protein and peptide identifications, enhancing biomarker discovery by providing information on protein abundance, modifications, and cancer‐related interactions [280]. Other essential proteomic databases and repositories include GPMDB, MassIVE, PASSEL, SRMAtlas, and Panorama utilizes for several studies including biomarker discovery [281]. MetaboLights offers small‐molecule profiles from various biological samples, enabling researchers to explore metabolic changes in cancer and identify metabolite biomarkers reflective of tumor metabolism and its influence on cancer progression [282].

### 5.4 Integrated Multiomics Databases

Integrated multiomics databases improve the simultaneous analysis of genomic, transcriptomic, proteomic, and metabolomic data, providing a thorough understanding of cancer biology. These platforms facilitate the integration of various data types, helping researchers identify biomarkers across different molecular layers. cBioPortal, a key open‐access platform, merges genomic, transcriptomic, and clinical data from large cancer studies like TCGA and ICGC, allowing for the investigation of mutations, expression patterns, and patient outcomes to uncover biomarkers. Another essential tool, Xenabrowser, offers interactive access to multiomics data from TCGA and ICGC, combining genomic, epigenomic, and transcriptomic data to identify clinically relevant biomarkers [23, 283].

## 6 Case Studies: Bioinformatics‐Driven Biomarker Discovery

Bioinformatics plays a vital role in identifying and validating key biomarkers in oncology, significantly impacting clinical decisions and personalized treatment strategies. This section features case studies that highlight successful biomarker discoveries enabled by bioinformatics workflows, emphasizing their therapeutic effects and contributions to advancements in targeted therapies within clinical oncology (Table 3).

**TABLE 3:** Bioinformatics‐driven cancer biomarker discovery: Identification, methodology, clinical significance, and applications.

Cancer	Biomarker	Discovery method	Clinical significance	Potential applications	References
BC	HER2 (ERBB2)	Gene amplification studies, FISH, IHC	Overexpression in 20–30% of breast cancers; associated with aggressive disease and poor prognosis	Targeted therapy with trastuzumab, pertuzumab; prognostic biomarker for treatment stratification	[284]
PC	PSA	Blood testing, ELISA	Elevated serum levels indicate prostate cancer presence; widely used for screening and prognosis assessment	Early diagnosis, monitoring therapy response, detection of recurrence	[285]
OC	CA‐125	Enzyme‐linked immunosorbent assay	Elevated levels associated with ovarian cancer; used to monitor disease progression and recurrence	Screening in high‐risk populations, monitoring therapeutic response, recurrence detection	[286]
LC	EGFR L858R mutations	PCR‐based sequencing, NGS	Activating mutations in EGFR are present in 10–15% of NSCLC, predictive of response to EGFR inhibitors like gefitinib	Predictive biomarker for targeted therapy with EGFR inhibitors (e.g., gefitinib, erlotinib)	[287]
CRC	KRAS G12V mutation	PCR‐based sequencing, NGS	Mutations in KRAS (in 40–50% of cases) predict lack of response to anti‐EGFR monoclonal antibodies	Identifying candidates for anti‐EGFR therapies (cetuximab, panitumumab)	[288]
Mel	BRAF V600E mutation	PCR‐based sequencing, NGS	Found in ∼50% of melanomas; associated with worse prognosis and response to targeted therapies like BRAF inhibitors	Predictive biomarker for BRAF inhibitors (e.g., vemurafenib, dabrafenib)	[289]
HCC	AFP	Blood test, immunoassays	Elevated AFP levels indicate HCC; also used to monitor treatment response and recurrence	Screening for HCC, monitoring therapy effectiveness, prognosis	[290]
PC	CA 19‐9	Enzyme‐linked immunosorbent assay	Elevated in pancreatic cancer, especially in advanced stages; linked to poor prognosis	Early diagnosis, monitoring disease progression, therapeutic response assessment	[291]
GC	MSI	PCR, NGS	MSI status indicates defective mismatch repair system; predictive of better response to immune checkpoint inhibitors	Predictive biomarker for immune therapy (pembrolizumab, nivolumab)	[292]
BLC	FGFR3 S249C mutation	PCR‐based sequencing, NGS	Found in 60–70% of nonmuscle‐invasive bladder cancers; associated with less aggressive tumors	Prognostic marker, target for FGFR inhibitors (e.g., erdafitinib)	[293]
Leuk	PML‐RARA fusion gene	Fluorescence in situ hybridization	Specific to acute promyelocytic leukemia; associated with abnormal proliferation and differentiation	Target for all‐trans retinoic acid (ATRA) and arsenic trioxide therapy	[294]
GBM	MGMT promoter methylation	Methylation‐specific PCR, pyrosequencing	Predictive of response to temozolomide chemotherapy; methylation inactivates MGMT gene and sensitizes tumors to chemotherapy	Prognostic marker, therapeutic guidance for chemotherapy decision‐making (temozolomide)	[295]
CC	HPV	PCR, hybrid capture, sequencing	Detection of high‐risk HPV genotypes (e.g., HPV16, HPV18) strongly associated with cervical carcinoma	Screening, vaccination, prevention, and monitoring recurrence	[296]
NSCLC	ALK rearrangements	FISH, RT‐PCR, NGS	ALK rearrangements (e.g., EML4–ALK) found in a subset of NSCLC; associated with sensitivity to ALK inhibitors	Targeted therapy with ALK inhibitors (e.g., crizotinib, alectinib)	[297]
MM	Beta‐2 Microglobulin	Blood test, immunoassays	Elevated levels associated with poor prognosis, correlates with tumor burden and kidney function	Prognosis assessment, monitoring disease progression, therapy evaluation	[298]
BC	BRCA1 185delAG, BRCA2 6174delT mutations	Genetic testing, NGS	Inherited mutations increase the risk of breast and ovarian cancers; predictive of response to PARP inhibitors	Risk assessment, predictive biomarker for PARP inhibitors (e.g., olaparib, talazoparib)	[299]

Abbreviations: FISH, fluorescence in situ hybridization; IHC, immunohistochemistry; HPV, human papillomavirus DNA; NGS, next‐generation sequencing; PCR, polymerase chain reaction; MSI, microsatellite instability; AFP, alpha‐fetoprotein; PSA, prostate‐specific antigen; NSCLC, non‐small cell lung cancer; BC, breast cancer; PC, prostate cancer; OC, ovarian cancer; LC, lung cancer; CRC, colorectal cancer; Mel, melanoma; HCC, hepatocellular carcinoma; PC, pancreatic cancer; GC, gastric cancer; BLC, bladder cancer; Leuk, leukemia; GBM, glioblastoma; CC, cervical cancer; MM, multiple myeloma; ELISA, enzyme‐linked immunosorbent assay; REF, references [333, 334, 335, 336, 337, 338, 339, 340, 341, 342, 343, 344, 345, 346, 347, 348].

### 6.1 Examples of Successful Biomarker Discovery

Numerous key biomarkers discovered through bioinformatics have transformed precision oncology. These biomarkers enhance cancer diagnosis and prognosis and guide therapies by identifying patients likely to respond to specific treatments. One significant example is PD‐L1 (programmed death‐ligand 1), which allows tumor cells to evade immune responses by attaching to the PD‐1 receptor [300]. Bioinformatics methods, including differential gene expression analysis and pathway enrichment, have played a vital role in identifying PD‐L1 as an immune checkpoint biomarker [301]. Examining high‐throughput RNA‐seq data with tools like DESeq2 and EdgeR have shown PD‐L1 upregulation in various cancers [21]. By merging transcriptomic data with clinical outcomes and immune‐related pathways through tools such as GSEA and IPA, researchers have gained a deeper understanding of PD‐L1's role in immune evasion [302]. PD‐L1 testing is instrumental in selecting patients who could benefit from immune checkpoint inhibitors like pembrolizumab and nivolumab, with bioinformatics crucial in associating PD‐L1 expression with responses to immunotherapy [300].

A significant breakthrough in biomarker research is the discovery of *BRCA1* and *BRCA2* germline mutations, which strongly indicate hereditary breast and ovarian cancers [303]. Breast and ovarian cancers are responsible for the majority of the cancer‐related deaths among women around the globe [304, 305, 306]. Bioinformatics analyses of extensive sequencing data have validated these mutations as highly penetrant biomarkers. Mutation detection tools, such as Mutect and GATK, were utilized to analyze whole‐exome sequencing (WES) and NGS data to identify *BRCA mutations* [307]. Additionally, variant annotation tools like ANNOVAR and SnpEff, combined with computational predictions, have assessed the functional implications of these mutations [308]. The clinical impact is significant as BRCA testing now serves a vital role in evaluating cancer risk and shaping treatment decisions, especially with PARP inhibitors like olaparib, which target *BRCA‐mutated* cancers [309]. Genetic testing for BRCA mutations has become the standard of care in oncology, particularly for patients diagnosed with breast and ovarian cancer [310].

### 6.2 Therapeutic Implications

The identification of predictive biomarkers significantly enhances our understanding of cancer biology and guides treatment decisions. These biomarkers enable the selection of targeted therapies, thereby facilitating customized treatments personalized to patients’ molecular profiles to attain optimal effectiveness.

*EGFR mutations* are associated with increased sensitivity to EGFR inhibitors such as erlotinib and gefitinib, especially in NSCLC [311]. Leveraging bioinformatic tools like VarScan and OncoKB is essential for identifying these mutations as actionable biomarkers [312]. By analyzing *EGFR mutations* via sequencing data, links to downstream pathways have been revealed, which assist in selecting targeted therapies and results in better clinical outcomes [313].

Rearrangements of anaplastic lymphoma kinase (ALK) in lung cancer have spurred the development of ALK inhibitors, such as crizotinib and alectinib [314, 315]. Bioinformatics tools, including FusionCatcher and STAR‐Fusion, evaluate RNA‐seq data for ALK fusions and network‐based tools like Cytoscape assist in validating ALK partners [316]. Testing for ALK rearrangements now determines eligibility for ALK inhibitors, thereby enhancing progression‐free survival rates in lung cancer [317].

TFF3 and pBAD are emerging as important targets in several malignancies including mammary, lung, liver, pancreatic, colorectal, ovarian, and endometrial due to their roles in tumor growth and treatment responses [318, 319, 320, 321, 322, 323, 324, 325, 326, 327, 328, 329, 330, 331, 332, 333, 334, 335, 336, 337]. They are vital for epithelial cell regeneration and are linked to poor patient outcomes. By targeting TFF3, it may be possible to hinder tumor development by influencing the tumor microenvironment [338]. Tools such as GEPIA and DAVID are useful for assessing TFF3 expression and its related pathways. Moreover, pBAD, recognized for its ability to promote apoptosis, could be harnessed to increase cell death in tumors, making them more susceptible to chemotherapy [327]. Analyzing TFF3 and pBAD expression through bioinformatics reveals their contributions to cancer progression and supports innovative treatment strategies.

## 7 Challenges in Bioinformatics‐Driven Biomarker Discovery

While bioinformatics has significantly transformed the process of biomarker discovery, challenges persist in translating computational results into clinically applicable biomarkers [339]. Principal issues include data integration, computational constraints, and the transition from discovery to clinical application. Addressing these challenges is crucial for improving bioinformatics methodologies and their impact on precision oncology.

### 7.1 Data Integration and Heterogeneity

One of the significant challenges in biomarker discovery driven by bioinformatics is integrating various omics data types—such as genomics, transcriptomics, proteomics, epigenomics, and metabolomics—into a unified framework [340]. Each omics layer offers unique insights while differing in structure, scale, and complexity; for example, genomic data are typically static, whereas transcriptomic and proteomic data are more dynamic. Advanced computational tools are required to tackle issues such as discrepancies in data types, noise, and missing values to effectively integrate these datasets. Despite continuous efforts using tools like iCluster and SNF, integrating multiomics data remain a significant challenge due to a lack of standardization, variations in sample collection methods, and disparate data processing pipelines [192, 340]. Additionally, the heterogeneity of cancer complicates biomarker discovery even further, as differences among patients and tumor subtypes imply that signatures may not be applicable across the board [262].

### 7.2 Interpreting Big Data

The massive amount of data produced by high‐throughput technologies poses a considerable challenge [341]. Current sequencing technologies generate terabytes of data, requiring advanced computational infrastructure for processing, storage, and analysis. Many bioinformatics tools demand significant computational power, which can be a barrier for researchers without access to high‐performance systems [342]. The necessity for real‐time analysis in clinical settings further increases the complexity. Proper long‐term storage of omics data, particularly in large cohort studies, requires robust data management systems, raising issues around data retrieval, sharing, and reproducibility. Additionally, the increase in data volume raises the likelihood of false positives and misleading correlations, especially in omics studies with multiple variables are simultaneously tested [343]. Addressing these challenges requires enhanced interdisciplinary collaboration and more effective validation and regulatory processes to facilitate the integration of bioinformatics‐driven biomarkers into clinical practice.

### 7.3 Translating Findings to Clinical Practice

Bioinformatics has significant potential for the discovery of biomarkers; however, applying these findings in clinical settings remains challenging [339]. Many biomarkers identified through bioinformatics do not transition to clinical practice due to issues with validation, regulatory hurdles, and the necessity to align computational findings with patient care. Comprehensive experimental validation, typically requiring large and well‐documented patient cohorts, is crucial for bioinformatics insights, yet such groups are frequently scarce [344]. Additionally, obtaining US FDA regulatory approval is a lengthy and expensive endeavor that involves multiple clinical trials. The gap between bioinformatics tools used for discovery and those available in clinical laboratories adds further challenges, as computational methods need to be reproducible, transparent, and pertinent for clinical application. This situation is exacerbated by insufficient collaboration between bioinformaticians and clinicians, hindering the effective prioritization of clinically significant discoveries. To overcome these obstacles, enhanced interdisciplinary collaboration and more efficient validation and regulatory processes are essential for successfully integrating bioinformatics‐derived biomarkers into clinical practice.

### 7.4 Clinical Integration of Omics Data in Precision Oncology

The integration of single‐cell and spatial omics data alongside clinical information represents a significant advancement in personalized cancer therapy [33]. This approach correlates molecular profiles with clinical outcomes, facilitating the discovery of new biomarkers and significantly enhancing the prediction of treatment responses. By utilizing various data sources, the researchers can generate actionable insights that directly inform targeted treatment strategies, ultimately resulting to more effective and customized patient interventions.

To fully utilize the potential of emerging technologies in precision oncology, it is crucial to pursue ongoing advancements in bioinformatics tools and analytical frameworks. Future research should focus on key areas such as integrating multiomics data, improving visualization techniques, and applying ML and AI [262]. Specifically, integrating multiomics data require creating advanced computational methods that combine information from genomics, transcriptomics, proteomics, and metabolomics, thus building a comprehensive model of cellular function and disease progression. This integrated approach deepens our understanding of cancer biology and helps identify clinically relevant biomarkers. Moreover, enhancing visualization capabilities is vital for interpreting high‐dimensional and complex datasets, making them more understandable and accessible for researchers and clinicians. By advancing visualization and computational methods, bioinformatics tools can yield deeper biological insights, improving patient stratification, diagnostic precision, and therapeutic decision‐making. Tackling these bioinformatics challenges will enhance our grasp of cancer mechanisms and accelerate the translation of multiomics findings into practical clinical applications, thereby driving the progress of personalized healthcare [23].

Researchers in precision medicine emphasize the necessity of standardized frameworks and forward‐looking clinical studies for ensuring reproducibility, quality assurance, and clinical relevance in bioinformatics methods [345]. By combining various data types, such as genomics, imaging, proteomics, and electronic health records (EHRs), there is a considerable opportunity to create and validate AI‐based medical models that improve predictive diagnostics, disease categorization, and treatment strategies [346]. These standardized frameworks enhance reproducibility by setting validated protocols for bioinformatics processes and AI models, while ensuring adherence to regulations and reliability in evaluations. Furthermore, incorporating diverse data strengthens the generalizability of AI, mitigates bias, and facilitates the transfer of computational insights into practical clinical applications. However, challenges remain, including the intricacy and heterogeneity of multimodal data, the requirement for comprehensive, well‐annotated clinical datasets, and the significant computational power needed for integration and model training [347]. Ethical and privacy issues regarding patient data also demand secure methods of sharing, like federated learning, to safeguard sensitive information while promoting collaborative research [269]. Additionally, regulatory challenges pose significant barriers to the validation and application of AI‐powered clinical decision‐support tools, making it necessary for collaborative efforts to standardize approaches across institutions and regulatory agencies [348].

Despite the existence of these challenges, addressing them through interdisciplinary collaboration among clinicians, bioinformaticians, data scientists, and regulatory experts is necessary to develop robust, interpretable, and clinically useful AI models [349]. While hurdles continue to persist, the advantages of precision medicine grounded in bioinformatics, such as enhanced patient stratification, biomarker identification, and personalized treatment options, are substantial. The ongoing innovation and advancement of these methodologies will continue to drive progress in cancer diagnostics and treatment strategies, ultimately transforming the future of personalized oncology care.

### 7.5 Ethical and Privacy Considerations in the Practical Application of Bioinformatics Tools in Precision Oncology

The integration of bioinformatics with AI and ML algorithms in clinical practice raises several vital ethical and privacy concerns [350]. Addressing these issues is crucial for protecting patient welfare, ensuring equitable access to care, and promoting the responsible use of advanced technologies. Ethical challenges in bioinformatics include data privacy, the importance of informed consent, data sharing, potential misuse of genetic data, the necessity for transparency in result interpretation, and algorithmic bias in AI and ML systems [351]. Establishing these ethical frameworks is critical for the effective use of biomarkers and designing personalized medicine while maintaining ethical standards.

Advanced sequencing and bioinformatics technologies produce massive amounts of genomic and clinical information, which risks confidentiality breaches if not properly safeguarded [352]. To preserve patient privacy and secure sensitive information, it is essential to anonymize or de‐identify data before sharing or publication [353]. However, the unique nature of genomic data raises concerns about achieving complete anonymization. This challenge requires the implementation of robust protocols and advanced computational tools to thwart reidentification attempts. Data encryption is another vital measure, ensuring safe transmission and storage of patient information [354]. Additionally, encryption tools help guard against unauthorized access by staff or external parties. To enhance these efforts, enforcing strict access controls, using secure servers, following regulatory frameworks like GDPR (General Data Protection Regulation) and HIPAA (Health Insurance Portability and Accountability Act), and allowing only authorized personnel access to sensitive information can significantly mitigate the risk of privacy violations [355].

Informed consent is a key ethical issue in biomedical research [352]. Patients whose data are used in such studies must receive detailed information regarding the study's purpose, the use of their biological samples and personal data, and any associated risks and benefits [356]. In bioinformatics, obtaining meaningful consent requires addressing the complexities of information sharing, broad consent, and the need for reconsent [357]. Effectively communicating complex genomic data and analysis to participants can be challenging. Thus, researchers should create explicit, accessible consent materials free of jargon, ensuring that participants fully understand the study's objectives and methodologies. Some researchers may opt for broad consent or move to dynamic consent, which supports long‐term scientific development and enables the future use of participants’ data in new research [358]. Furthermore, this approach can enhance scientific exploration, while necessitating a strong ethical commitment to respect participants’ autonomy, including their right to withdraw consent at any moment during the study.

Alongside privacy concerns, sharing data and ensuring equitable access to precision oncology studies and analyses are crucial [359]. Data sharing is vital in bioinformatics but requires solid agreements to maintain ethical standards and protect participants' rights [360]. Repositories such as dbGap (database of genotypes and phenotypes) offer dual access: open‐access data for transparency and a controlled‐access system for participant privacy protection [361]. Controlled access allows researchers to responsibly use sensitive data while ensuring participant confidentiality, thus aligning the benefits of shared data with ethical responsibilities. Precision oncology must ensure that innovations based on biomarkers are available to all patient demographics, including those from low socioeconomic or underrepresented backgrounds [362]. Social justice should guide funding and resource allocation decisions to prevent healthcare disparities. Additionally, predictive models, including AI and ML algorithms, play a vital role in bioinformatics, yet they also raise significant ethical issues that require careful consideration [363]. A key problem with AI systems is the biases inherent in training data, which can involve missing information, unrecognized patients (algorithmic bias), and sample size challenges and misclassification. For example, vulnerable populations, such as those with low socioeconomic status, psychosocial obstacles, and immigrants, often experience nonrandom data omissions in healthcare systems, leading to gaps in EHRs (e.g., absence of diagnostic tests, chronic illness medications, or social factors like housing instability) [351, 363]. Such deficiencies can lead ML algorithms to misinterpret existing data or exclude at‐risk individuals from clinical tools designed for early intervention. Furthermore, EHRS might miss data on specific elements critical for enhancing health outcomes in these populations. Tackling these biases necessitates employing fairness‐aware algorithms, utilizing diverse training datasets, and intentionally integrating metadata to prevent discriminatory treatment outcomes that could worsen socioeconomic healthcare disparities or disproportionately affect certain groups [363, 364]. Key steps include identifying or defining the target population, choosing training/testing datasets that embody this diversity, developing and validating algorithms across varied healthcare contexts, rigorously examining potential discriminatory patterns during data processing, establishing feedback mechanisms for validating output, and prioritizing clinically meaningful results instead of just performance benchmarks. These strategies are intended to promote equitable healthcare delivery and ensure that ML‐based algorithms and tools effectively serve all populations, especially those typically marginalized in data representation [360].

The ethical aspects of bioinformatics are complex, influenced by technical details and societal duties. A key concern is the lack of clarity in AI models, which can hide decision‐making processes. It is crucial to ensure transparency and accountability in AI‐driven decisions to uphold trust, particularly given bioinformatics’ impact on research outcomes. Additionally, ethical considerations require careful attention to essential elements of ethical bioinformatics practices, including data privacy, informed consent, and responsible data sharing, alongside measures to prevent the misuse of participants’ genetic information [352, 360].

## 8 Future Directions

The prospective trajectory of bioinformatics research in precision oncology holds transformative possibilities, fueled by progress in computational power, AI, ML, and collective data‐sharing platforms. Significant advancements are driven by refined computational methods, the integration of AI and ML, and strengthened collaboration to establish a cohesive framework for discovering biomarkers and strategies in precision medicine [365].

With the increasing reliance on data in cancer research, the future of biomarker discovery and therapeutic advancements depends on utilizing advanced computational methods and state‐of‐the‐art technologies. Scalable cloud computing solutions like AWS, Google Cloud, and Microsoft Azure, coupled with distributed systems for parallel processing, will significantly enhance the efficiency of large‐scale omics data analysis. User‐friendly tools such as Galaxy, Seven Bridges, and DNAnexus are poised to optimize complex data workflows, while innovative single‐cell technologies and spatial omics approaches offer exceptional insights into cellular dynamics and the tumor microenvironment, opening new avenues for discovering biomarkers and drug targets [366].

Rapid advancements in ML and AI are transforming the future of precision oncology [367]. Advanced deep learning models, utilizing multiomics datasets, are anticipated to uncover new biomarkers, predict responses to treatment, and identify actionable therapeutic targets with greater precision. By integrating AI into clinical workflows, oncologists will be equipped to make personalized, data‐driven treatment choices that reflect each patient's molecular profile [368]. Additionally, the role in enhancing prevention, screening, and prognostic predictions for cancers holds significant potential for early intervention and better patient outcomes. Collaborative efforts and open data‐sharing approaches are critical for progressing cancer research. Public databases such as TCGA, ICGC, and GEO will continue to enable cross‐cohort validation and reproducibility, while initiatives like the Global Alliance for Genomics and Health and ELIXIR will develop strong data‐sharing standards [369, 370]. Platforms like cBioPortal and XenaBrowser are poised to improve data visualization and collaborative research, encouraging cooperation among academic scientists, industry innovators, and healthcare professionals [283, 371].

In the future, the integration of advanced computational technologies, AI/ML advancements, and collaborative environments will transform biomarker discovery and treatment strategies in precision oncology. These innovations will significantly enhance our understanding of cancer biology, increase the reliability of biomarkers, and enhance patient outcomes with more effective, personalized therapies, leading to a transformative phase in cancer care.

## 9 Limitations of Bioinformatics Tools in Precision Oncology

Bioinformatics tools utilized across diverse omics levels in precision oncology face many limitations that negatively impact their effectiveness in biomarker discovery [16]. Genomic instruments like DESeq2 for gene expression analysis and GATK for variant calling require considerable computational resources and show performance variability, especially at extreme expression levels. Similarly, functional genomics tools such as GSEA and KEGG often struggle with context specificity due to differing pathway relevance, which is affected by gene annotations and the limitations of related databases.

Epigenomic tools, such as Bismark for DNA methylation and MACS for chromatin accessibility are sensitive to sequencing errors. They face challenges in repetitive regions and situations with low‐signal data. Similarly, proteomics and metabolomics tools, such as MaxQuant and XCMS, struggle to detect low‐abundance analytes and rely on high‐quality reference libraries. On the other hand, lipidomics tools like LipidSearch require specialized standards to manage the complexity of lipid diversity effectively [31, 372].

Transcriptomic tools for both bulk and single‐cell RNA‐seq, including Salmon and Seurat, encounter difficulties due to noise and dropout rates, which hinder accurate quantification and annotation [373]. Integrative multiomics tools are crucial for combining diverse data but demand significant computational resources and sophisticated interpretation for effective integration. Furthermore, cancer‐specific tools such as cBioPortal and TIMER, which play a crucial role in biomarker identification and immune infiltration analysis, often struggle with limited dataset availability. They may also miss rare mutations and tumor heterogeneity, key factors essential for personalized medicine oncology [23].

## 10 Conclusion

Bioinformatics is crucial for discovering biomarkers and developing therapeutic strategies in precision oncology by allowing the analysis of intricate, high‐throughput datasets [16]. By integrating multiomics data—including genomics, transcriptomics, proteomics, epigenomics, and metabolomics—researchers can decipher molecular mechanisms underlying cancer and identify clinically significant biomarkers for diagnosis and prognosis. For example, genomic biomarkers such as *EGFR* and *BRCA1/2* mutations guide targeted therapies like *EGFR* inhibitors (erlotinib, gefitinib) for NSCLC and PARP inhibitors (olaparib, niraparib) for *BRCA1/2*‐mutated breast and ovarian cancers [14, 374]. Additionally, epigenetic biomarkers like *MLH1* methylation assist in optimizing treatment selection [12]. Advances in these areas have also enhanced immunotherapy, with immune checkpoint inhibitors (pembrolizumab, nivolumab) targeting the *PD‐1/PD‐L1* axis to enhance antitumor responses [375]. HER2‐targeted therapies, such as trastuzumab and pertuzumab, have been instrumental in treating HER2‐amplified breast and gastric cancers by blocking HER2 signaling and inhibiting tumor growth [376].

In addition to single‐agent therapies, bioinformatics insights have facilitated the development of combination strategies that enhance efficacy by simultaneously targeting multiple pathways [377]. For example, pembrolizumab is administered with chemotherapy for NSCLC, trastuzumab is combined with pertuzumab for HER2‐positive breast cancer, bevacizumab is combined with chemotherapy for metastatic colorectal cancer, and bortezomib is used with dexamethasone for multiple myeloma [378, 379, 380, 381]. These cotargeting methods utilize network‐based analyses to enhance treatment regimens and improve clinical outcomes. The transformative role of bioinformatics is further strengthened by ML and AI, which automate complex data analyses, uncover hidden patterns, and enhance predictive biomarker discovery [382]. For instance, the overexpression of secreted oncoproteins like human growth hormone (*hGH*) and trefoil factor family (*TFF*) proteins has been recognized as a significant factor in cancer progression through ML‐based pattern recognition [328]. Integrating multiomics platforms and employing network‐based modeling has facilitated the discovery of robust biomarkers across various molecular layers and informed therapeutic strategies by connecting these biomarkers to actionable pathways [383]. An illustrative example is the use of synthetic lethality‐based approaches to exploit weaknesses in DNA repair pathways with PARP inhibitors (olaparib, rucaparib, niraparib, and talazoparib) in cancers harboring *BRCA* or *ATM* mutations [384]. Additionally, emerging technologies such as cloud computing, distributed systems, single‐cell analysis, and spatial omics continue to enhance tumor profiling and heterogeneity evaluation [385, 386]. Platforms like TCGA and cBioPortal harness cloud‐based bioinformatics, while FireCloud supports distributed computing for extensive data analysis [283]. Techniques like single‐cell RNA‐seq (*scrna‐seq*) and spatial transcriptomics (e.g., CyTOF) enable detailed tumor mapping, allowing for the observation of cellular interactions within the tumor microenvironment [387].

Looking ahead, ML and AI are expected to transform biomarker discovery and therapeutic design by improving our understanding of tumor evolution, drug response, and resistance mechanisms. With the rise of collaborative efforts and open data‐sharing initiatives, the clinical application of bioinformatics‐driven discoveries is expected to accelerate, ultimately advancing precision oncology through the development of more effective, personalized therapies for patient care.

## Author Contributions

T.W., V.B., and V.P. contributed to the conceptualization, data curation, and formal analysis of the review. T.W. and V.B. were responsible for writing the original draft preparation, visualization, and methodology. V.P. reviewed and edited the manuscript. V.P. provided supervision, project administration, and resources and secured funding for the study. All the authors have read and approved the final version of the manuscript.

## Conflicts of Interest

The authors declare that this research was conducted in the absence of any commercial or financial relationships that could be construed as potential conflicts of interest.

## Ethics Statement

The authors have nothing to report.

## Data Availability

The data that support the findings of this study are openly available in toothgennet‐anterior at https://github.com/superbijk/toothgennet‐anterior/.
